# Preparation and Characterization of Photo-Cross-Linkable Methacrylated Silk Fibroin and Methacrylated Hyaluronic Acid Composite Hydrogels

**DOI:** 10.1021/acs.biomac.4c00319

**Published:** 2024-10-14

**Authors:** Jhaleh Amirian, Jacek K. Wychowaniec, Matteo D′este, Andrea J. Vernengo, Anastasija Metlova, Antons Sizovs, Agnese Brangule, Dace Bandere

**Affiliations:** † Department of Pharmaceutical Chemistry, Riga Stradins University, Riga LV-1007, Latvia; ‡ Baltic Biomaterials Centre of Excellence, Headquarters at Riga Technical University, Riga LV-1048, Latvia; § AO Research Institute Davos, Clavadelerstrasse 8, Davos 7270, Switzerland; ∥ Laboratory of Pharmaceutical Pharmacology, Latvian Institute of Organic Synthesis, Riga LV-1006, Latvia

## Abstract

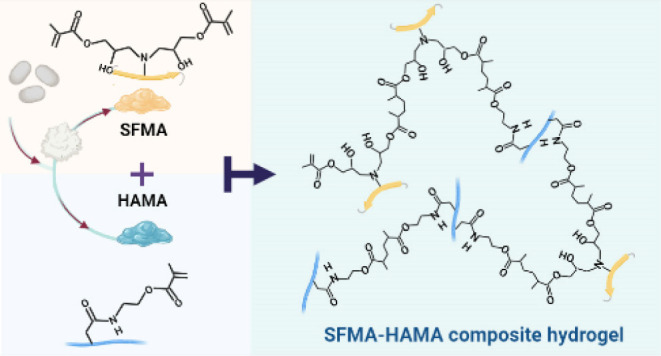

Composite biomaterials with excellent biocompatibility and biodegradability are crucial in tissue engineering. In this work, a composite protein and polysaccharide photo-cross-linkable hydrogel was prepared using silk fibroin methacrylate (SFMA) and hyaluronic acid methacrylate (HAMA). SFMA was obtained by the methacrylation of degummed SF with glycidyl methacrylate (GMA), while HA was methacrylated by 2-aminoethyl methacrylate hydrochloride (AEMA). We investigated the effect of the addition of 1 wt % HAMA to 5, 10, and 20 wt % SFMA, which resulted in an increase in both static and cycling mechanical strengths. All composite hydrogels gelled under UV light in <30 s, allowing for rapid stabilization and stiffness increases. The biocompatibility of the hydrogels was confirmed by direct and indirect contact methods and by evaluation against the NIH3T3 and MC3T3 cell lines with a live–dead assay by confocal imaging. The range of obtained mechanical properties from developed composite and UV-cross-linkable hydrogels sets the basis as possible future biomaterials for various biomedical applications.

## 1 Introduction

In recent years, various types of hydrogels based on natural and synthetic polymers, which employed chemically driven cross-linking mechanisms, were developed.^1,2^ In particular, photo-cross-linkable hydrogels have received a great deal of attention due to the fact that light can be used to control the reaction pattern of the material in a fine spatiotemporal manner, or to stabilize the printed structures and increase their fidelity.^3−5^ It has been shown that the appropriate choice of chemical cross-linking approach leads to the generation of hydrogel networks with properties tailored to the desired clinical or tissue engineering application.^6^ Among all types of hydrogels, peptide-, protein- and polysaccharide-based hydrogels have been widely studied as biocompatible matrices for numerous biomedical applications.^7−9^

There are several challenges to the development of biocompatible hydrogels for tissue engineering.^10^ As a major challenge, it is important to ensure that the biomaterial mimics the native extracellular matrix (ECM).^11^ Furthermore, many existing hydrogels do not have the required mechanical strength, biocompatibility, or the ability to promote the growth and differentiation of cells.^12^ Therefore, it is imperative to develop new biomaterials that are capable of overcoming these limitations in order to provide enhanced performance in specific applications such as tissue engineering, wound healing, and drug delivery. Various hydrogels including polysaccharide and protein-based ones were extensively used in the field of tissue engineering and biomedicine.^13^ However, these hydrogels, in their monophasic state, exhibit deficiencies in their mechanical strength and biocompatibility. Consequently, these constraints impede their utilization in biomedical contexts. However, according to the literature, combining proteins and polysaccharides can result in synergistic delivery systems with improved mechanical strength and enhanced biocompatibility.^13^ These composite hydrogels offer significant performance improvements over monophasic hydrogels through the use of unique properties including printability, mechanical strength, structural improvements, and biocompatibility.^14^

In particular, silk fibroin (SF), a protein-based biopolymer, shows good biocompatibility, adjustable biodegradability, and mechanical strength and low immunogenicity.^15^ The SF scaffolds are robust and allow them to withstand mechanical stress, making them suitable for load-bearing applications, such as cartilage tissue engineering and bone tissue engineering. In addition, advantages such as abundant sources, ease of production and availability, low immunogenicity, and high safety make them suitable for hydrogel formation as a versatile tissue engineering support.^16^ SF is obtained from a variety of sources, including silkworms, spiders, and mites.^17^ The SF extract of silkworm silk (*Bombyx mori*) is composed of highly repetitive primary peptide sequences GAGAGS, which have received considerable interest in biomedicine.^6,18^ In our recent review, we pointed out the role of the chemical routes for the generation of functional SF methacrylate (SFMA) using different methacrylation agents.^6^ Glycidyl methacrylate (GMA) was found to be the most effective methacrylation agent since it reacts with both amine and hydroxyl groups and does not produce any byproducts such as methacrylic acid.^6^

Another noteworthy biopolymer is hyaluronic acid (HA), which is one of the major glycosaminoglycans that build multiple tissues.^18−20^ A large proportion of HA can be found in the ECM of higher animals, and it plays a critical role in cell differentiation and cell motility.^18−25^ HA and its various chemically functionalized forms (including methacrylated, or tyraminated) have been successfully used as biomaterials and bioinks.^18,19,21−24^

Grafting the monomer with the double bond onto the polymer chain of the hydrogel, SF and HA, can result in a photo-cross-linkable hydrogel precursor that can serve as an ink for 3D printing.^3,26−28^ Most frequently, methacrylation agents like methacrylic anhydride (MA),^6,12,26,29^ GMA,^6,27,28^ 2-isocyanatoethyl methacrylate (IEM),^6,30^ and 2-aminoethyl methacrylate hydrochloride (AEMA)^31^ are used to functionalize polymers with methacryloyl groups in order to make them photo-cross-linkable. Through free-radical chain polymerization in the presence of UV and light, these methacrylated precursors were cross-linked effectively in a short period of time, approximately <1 min at a wavelength range of 320–500 nm and a variable power intensity ranging from 5 to 2500 mW/cm^2^.^6,32^ The degree of methacrylation,^6,27^ concentration of the polymer,^6,27^ photoinitiator (PI),^6,27,33^ and light intensity^6,27^ could all be adjusted to alter the chain polymerization and amend the final properties of the cross-linked hydrogels.

Based on previous approaches to synthesize HAMA, in this work, we devised an alternative approach to methacrylate HA using AEMA.^34^ Furthermore, we synthesized SFMA using GMA, similar to previous studies.^27,28,37^ We then investigated the structural, physiochemical, rheological, and mechanical properties, as well as cell viability and biocompatibility, of HAMA, SFMA, and their composites SFMA/HAMA as a function of SFMA content in order to generate a family of functional hydrogels and inks for future biofabrication and tissue engineering studies.

## 2 Materials and Methods

### 2.1 Chemicals

*Bombyx mori* cocoons (Mainland China), sodium carbonate (Na_2_CO_3_), sodium chloride (NaCl), glycidyl methacrylate (GMA, ≥97%, Sigma-Aldrich), hyaluronic acid sodium salt (HA, *M*_W_ = 290 kDa, 5 mM carboxylic groups; Contipro Biotech S.R.O), polyethylene glycol (PEG, *M*_W_ = 20 kDa, Sigma-Aldrich), lithium bromide (LiBr, ≥99%, Sigma-Aldrich), polyethylene glycol (PEG, *M*_W_ = 20 000, Sigma-Aldrich), 2-(*N*-morpholino)ethanesulfonic acid (MES buffer, ≥99%, Sigma-Aldrich), 1-ethyl-3-(3-dimethylaminopropyl)-carbodiimide hydrochloride (EDC, ≥98%, Sigma-Aldrich), *N*-hydroxysuccinimide (NHS, ≥98%, Sigma-Aldrich), and 2-aminoethyl methacrylate hydrochloride (AEMA, ≥90%, Sigma-Aldrich) were purchased and used as-received.

*In vitro* studies were conducted using NIH3T3 and MC3T3 cells obtained from the American Type Culture Collection (ATCC, USA). In order to conduct the *in vitro* study, Dulbecco’s modified Eagle medium (DMEM), fetal bovine serum (FBS, Gibco, Thermo Fisher Scientific), penicillin–streptomycin (PS, Gibco, Thermo Fisher Scientific), phosphate-buffered saline 1× (PBS, Gibco, Thermo Fisher Scientific), isopropyl alcohol (Sigma-Aldrich), and MTT (3-(4,5-dimethylthiazol-2-yl)-2,5-diphenyltetrazolium bromide) (BLD Pharmatech LTD, Shanghai, China) were purchased and used as received.

### 2.2 Methods

#### 2.2.1 Degumming

SF was produced by adapting previous protocols.^35,36^ Namely, 6.25 g of *Bombyx mori* cocoons were cut into pieces of approximately 5 mm × 5 mm and then boiled in 2.5 L of Milli-Q water containing 5 wt % sodium carbonate for 35 min to degum them. Fibers were then rinsed through immersion in distilled water and subsequently pressed to release the absorbed liquid for 3–5 times, while being stirred for 30 min.^35^ The process was repeated five times to eliminate glue-like proteins (sericin and wax) from the samples. Finally, the fibers were squeezed to release all liquid and then placed on aluminum foil under a fume hood at room temperature until they became fully dry.^35^

#### 2.2.2 Methacrylation of SF by GMA

**Figure 1:**
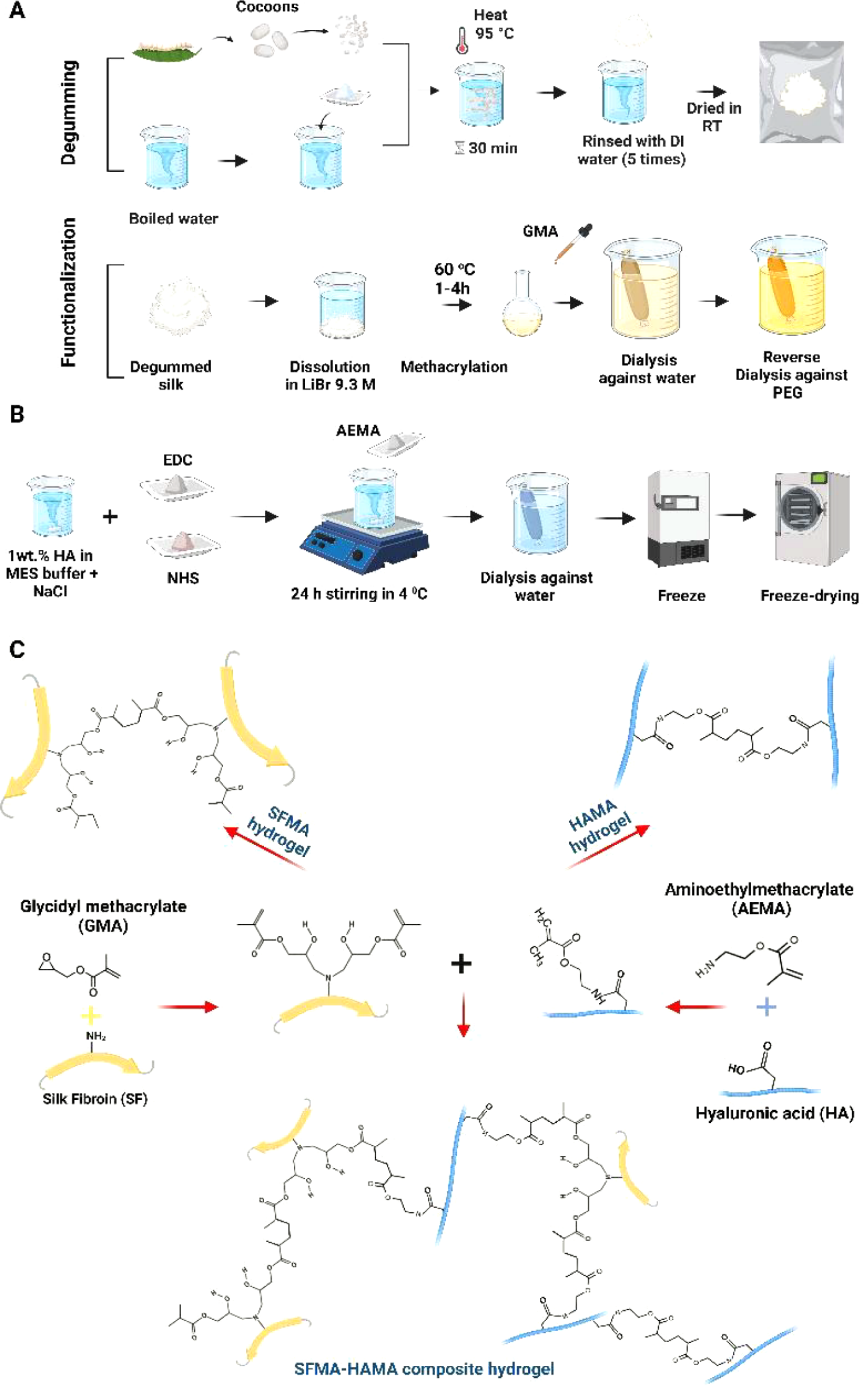
An overview of SF extraction and SFMA and HAMA synthesis, including (A) silk degumming; dissolution of SF in LiBr; and methacrylation, dialysis, and concentrating of the SFMA; (B) methacrylation of the HA by AEMA; and (C) a photo-cross-linking reaction using free radical vinyl polymerization at 365 nm to obtain the SFMA, HAMA, and SFMA–HAMA hydrogels. Figure created with Biorender.com.

Methacrylation of SF was performed by adapting previous protocols,^27,28,37^ with minor modifications. Briefly, a total of 2.2 g of degummed SF (D-cocoon) was dissolved in 17.6 mL of 9.3 M LiBr solution (8-fold the amount of dried degummed SF) and kept at 60 °C for 4 h. We successfully dissolved the SF in the LiBr solution and then slowly added 2 mL of GMA and stirred it at 300 rpm for 3 h at 60 °C to achieve a DoM of 41.6 ± 4.5% under comparable conditions with the preceding study done by Kim et al., who achieved 42%.^27^ After the SF was successfully dissolved in the LiBr solution, 2 mL of GMA was slowly added and stirred at 300 rpm for 3 h at 60 °C. As part of the purification process, the solution was dialyzed against distilled water *via* 12–14 kDa cut-off cellulose membranes for 60 h at room temperature. During the first and fourth hour of dialysis, there were two changes in water, followed by six changes within the next 2 days. Since SF and SFMA are sensitive to temperature,^38,39^ the initial dialysis procedure was followed by an additional 5 days of dialysis at 4 °C for complete purification. The SFMA solution was collected in a 50 mL conical tube and centrifuged for 20 min at 9000 rpm in 4 °C, and the process was repeated three times in total. The supernatant solution was filtered through a 100 μm strainer and kept in fresh conical tubes at 4 °C. To determine the concentration, 0.5 mL of the SF solution was placed in a weighing boat and kept at 60 °C for several hours to determine its weight gravimetrically. When required, to yield higher concentrations, the SF solution was reverse-dialyzed against 10 wt % of 20 kDa PEG for several hours until the desired concentration was reached. This process is depicted in Figure 1A, which shows the extraction of SF and its subsequent modification with GMA.

#### 2.2.3 Methacrylation of HA by AEMA

In this study, the methacrylation of hyaluronic acid (HA) was conducted through an adapted reaction method initially proposed by Mehrali et al.,^31^ wherein pectin was substituted with hyaluronic acid, and AEMA was utilized as the methacrylation agent. The first step involved preparing 100 mL of MES buffer solution with a concentration of 0.05 M and pH 6.5 and with 0.5 M NaCl. Next, 1 g of HA was added to the solution and stirred at 4 °C. Then, NHS and EDC powders, each with a concentration of 0.06 and 0.12 M, respectively, were added to the completely dissolved HA solution. Then, AEMA powder at a total concentration of 0.06 M was added to the solution after 30 min and allowed to react with HA at 4 °C for 24 h. The concentrations of MES, EDC, NHS, and AEMA have been calculated based on the initial volume of the solution. This followed a molar ratio of 1:2:1 (NHS:EDC:AEMA) used to synthesize HAMA. Upon completion of the reaction, a solution was dialyzed against a 12–14 kDa cut-off dialysis tubing for 7 days at 4 °C. As a final step, the solution was frozen overnight at −80 °C and subsequently lyophilized over 7 days. A diagram illustrating this synthesis approach can be found in Figure 1B.

#### 2.2.4 Hydrogel Preparation and UV Cross-Linking

The SFMA, HAMA, and SFMA–HAMA hydrogels were fabricated with mass ratios shown in Table 1. To construct photo-cross-linked SFMA (5, 10, and 20 wt %), a concentrated solution of synthesized SFMA was diluted and subsequently mixed with LAP at a concentration of 0.4 wt % at room temperature, until the final desired concentrations were reached. The choice of photoinitiator type and concentration has been made on the basis of established protocols and proven results from the established protocols.^37,40^ To prepare composite hydrogels, SFMA5–HAMA1, SFMA10–HAMA1, and SFMA20–HAMA1, HAMA solution at 4 wt % was first mixed with LAP and subsequently with the concentrated SFMA. Figure 1C illustrates the preparation of the SFMA, HAMA, and SFMA–HAMA hydrogels. For certain characterizations, photo-cross-linked hydrogels were prepared by pouring the desired hydrogel solution into PDMS molds with well sizes of 10 mm × 4 mm and exposition to UV light for 3 min at 7 mW/cm^2^ radiation power.

**Table 1:** List of Prepared Hydrogel Samples and their Abbreviations

Sample	HAMA [wt %]	SFMA [wt %]
HAMA1	1	0
SFMA5	0	5
SFMA10	0	10
SFMA20	0	20
SFMA5–HAMA1	1	5
SFMA10–HAMA1	1	10
SFMA20–HAMA1	1	20

### 2.3 ^1^H-Nuclear Magnetic Resonance (^1^H NMR)

To confirm methacrylation, Bruker Avance III 300 MHz NMR was used to measure the ^1^H NMR spectra for HA and HAMA in D_2_O and SF and SFMA in LiBr solution with 9.3 M concentration in D_2_O, all at concentrations of 10 mg/mL. The degree of methacrylation (DoM) of SFMA and HAMA biopolymers was calculated following the previously published methods.^27,41,42^ Four different batches were analyzed to assess the DoM percentage for both HAMA and SFMA. The peaks were integrated after baseline correction. According to the following equation, the DoM was calculated for SFMA.^27,41^
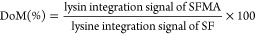
1

The DoM of HAMA was qualitatively determined by comparing the integration of vinyl protons at 5.76 and 6.16 ppm with the integration of methyl protons at 2.15 ppm, as described by eq 2.^42^
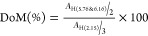
2

**Figure 2:**
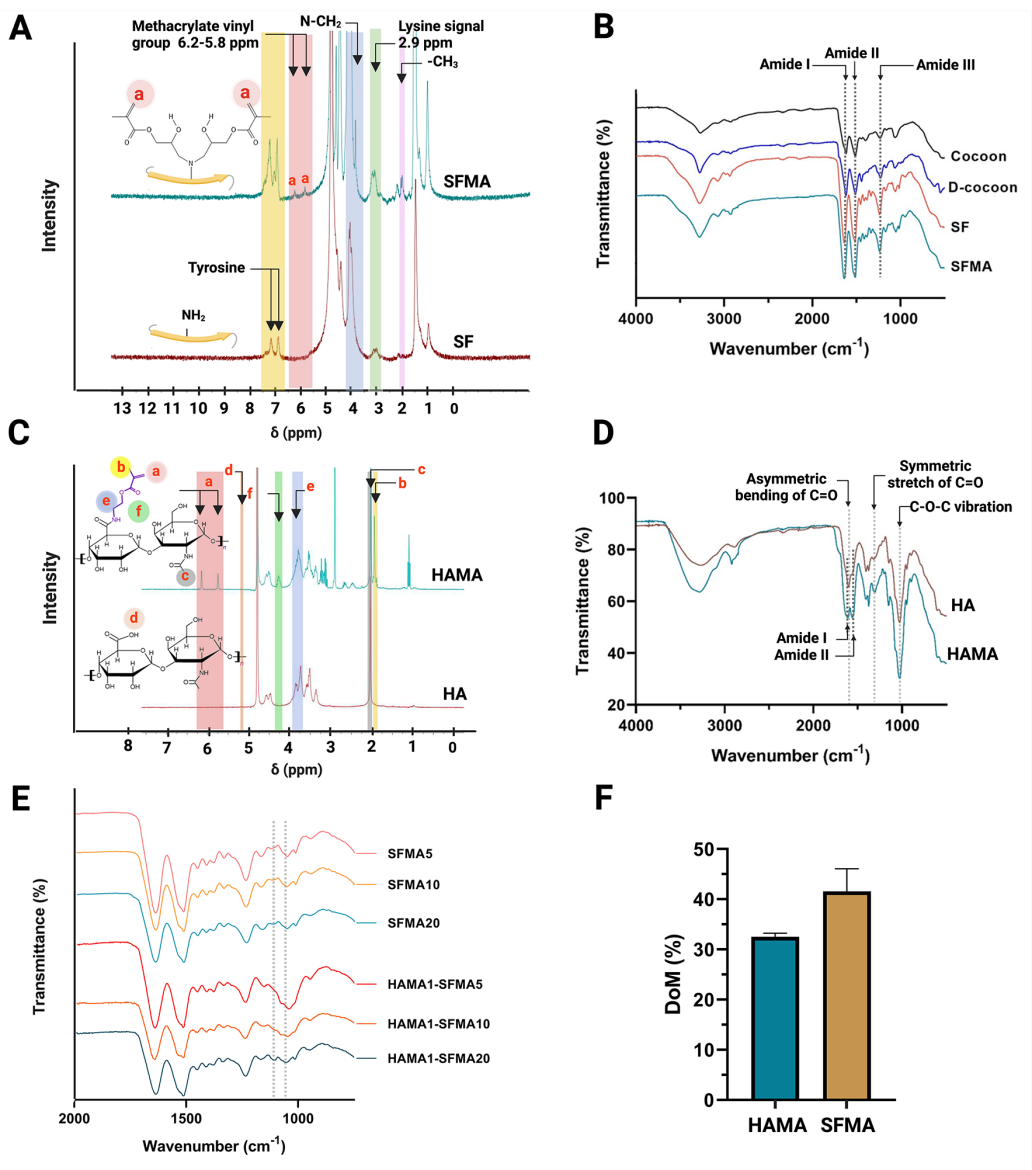
(A) ^1^H NMR spectra of the SF and SFMA. The presence of the methacrylate vinyl group signal with DoM of 41.6 ± 4.5% (calculated as described in methods), yellow aromatic amino acid resonances (6.7–7.5 ppm) that are not chemically modified, pink methacrylate vinyl groups that appear after the reaction (5.6–6.5 ppm), and green lysine amino acids (2.9–3.0 ppm). (B) FTIR spectra of BM cocoon, D-cocoon (degummed cocoon), SF, and SFMA, (C) ^1^H NMR spectra of HA before and after modification with AEMA. The presence of the methacrylate vinyl group signal with DoM of 32.5 ± 0.7% (calculated as described in methods), pink methacrylate vinyl groups that appear after the reaction amine group of AEMA with COOH groups of the HA (5.7 and 6.1 ppm), orange COOH that appear (5.2 ppm), yellow and grey methyl groups that appear (1.9 and 2.1 ppm), and purple and green methylene group that appear (3.7 and 4.2 ppm). (D) FTIR spectra of HA and HAMA. (E) FTIR spectra of SFMA and SFMA–HAMA hydrogels after UV cross-linking. (F) DoM graph of SFMA and HAMA hydrogels.

Here, *A*_H(5.76&6.16)_ and *A*_H(2.15)_ represent the areas around mean intensities of vinyl protons (^1^H of part a noted in molecule inset scheme in Figure 2C) at 5.76 and 6.16 ppm and the area around mean intensity of methyl proton (^1^H of part d noted in molecule inset scheme in Figure 2C) at 2.15 ppm, respectively.

### 2.4 Fourier Transform Infrared Spectroscopy (FT-IR)

The FTIR analysis was performed within the wavelength range of 400–4000 cm^–1^ using a Nicolet iS50 spectrometer (Thermo Fisher Scientific, USA), with a resolution of 4 cm^–1^ and 64 scans applied for each sample. Prior to the collection of the sample spectrum, an air background spectrum with no sample was acquired. The deconvolution was implemented in OriginLab by employing a Gaussian model. The percentage of secondary structures was determined by utilizing the single band areas of the deconvoluted amide I spectra (1600 cm^–1^ to 1700 cm^–1^). The assignment of the peak band was based on the findings of Hu et al.^43^

### 2.5 Scanning Electron Microscopy (SEM)

Scanning electron microscopy (SEM, TESCAN VEGA, Czech Republic) was performed to analyze the morphology of lyophilized hydrogels at a 5 keV acceleration voltage. After the samples were transferred to the SEM stage, they were sputtered with 15 pulse mode carbon nanotubes. The pore diameters of 20 randomly selected areas from five images were determined by using ImageJ software.

### 2.6 Rheology

The rheological measurements were performed on an MCR302 rheometer (Anton Paar). Parallel plate geometry with a 25 mm diameter plate was used. A gap of 0.5 mm was used in the oscillatory settings (amplitude and gelation/time sweeps), and that of 1 mm was used in rotational mode (flow curve). All measurements were carried out at 37 °C. *In situ* gelation by UV cross-linking was measured using a quartz crystal stage and a UV light source (Omnicure 1000, Lumen Dynamix LDGI) illuminating the underside of the deposited sample. Amplitude sweeps in oscillatory mode were performed at a constant frequency of 1 Hz with strain increasing from 0.01 to 1000%. UV photo-cross-linking was performed in a time-sweep setting at a frequency of 1 Hz and 0.2% strain falling within the linear viscoelastic region (LVR) for 10 min. Flow curves were performed on hydrogels pre-cross-linked for 2 min on the stage and then subjected to an increasing shear rate from 0.1 and 1000 s^–1^.

### 2.7 Static and Dynamic Compression Testing

Hydrogel samples were prepared in the same manner as described before in Section 2.2.4. The solutions were placed in PDMS molds and exposed to UV light for 3 min at 7 mW/cm^2^ radiation power in cylindrical shapes of 5 mm height and 15 mm diameter. Between the tests, samples were kept in PBS at 4 °C, and immediately prior to the measurements, the PBS was gently blotted with tissue. In this study, uniaxial compression tests were conducted on an electrodynamic testing system (LTM1, ZwickRoell) equipped with a load cell of 50 N (U9C, HBM). The testing protocol consisted of a preloading of 0.02 N for SFMA5, HAMA1, and SFMA5–HAMA1 or 0.05 N for SFMA10, SFMA20, SFMA10–HAMA1, and SFMA20–HAMA1, followed by a compression to a maximum of 60% strain (3 mm) at a rate of 1 mm/min (0.33%/sec). The data were acquired at a sampling rate of 50 Hz. Following the plotting of stress–strain graphs, the linear portion of the curves was used to calculate Young’s modulus for each sample. The dynamic compression testing protocol was composed of the following steps: 0.05 N for all the samples, followed by setting a peak value at the static compression of 10% strain (0.5 mm). Subsequently, a sine wave was applied with a modulation of 5% amplitude (i.e., 0.25 mm up and down) for 1 h at 1 Hz, i.e., 3600 cycles in total. Data acquisition was performed at a sampling rate of 50 Hz. Stress–strain graphs were then plotted, and Young’s modulus for each sample was calculated from the linear portion of the obtained curves. For all tests, 3 independently prepared samples (*n* = 3) were measured. Figure 6A presents the diagram of the static mechanical measurement setup, while Figure 6D illustrates the dynamic mechanical analysis (DMA) process and its underlying working principle. Additionally, Figure 6E provides a schematic outlining the method used to calculate the energy dissipation efficiency (EDE) (%) across various cycles (1, 900, 1800, 2700, and 3600 cycles).

### 2.8 Swelling and Degradation Studies

To analyze swelling and degradation of the prepared hydrogels, HAMA, SFMA, and SFMA–HAMA hydrogels (10 mm × 4 mm) were soaked in PBS in time intervals of 1, 2, 3, 4, 5, 6, 18, and 24 h at 37 °C. Initially, a dry hydrogel was weighed (*W*_0_); after that, 2 mL of PBS was added, and afterward, degradation/swelling studies were started by putting gels immediately to an incubator and kept at 37 °C. Within the determined time intervals, the weight of the swollen hydrogels, *W*_t_, was recorded until the equilibrium had been reached. Using eq 3, the swelling percentage was calculated.
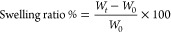
3

*In vitro* hydrogel degradation was carried out in accordance with ASTM F1635-16 and other prior studies.^1,44−46^ Weight changes in samples immersed in PBS at 37 °C within the designated time intervals of 7, 14, 21, 28, 35, 42, 49, 56, 63, and 70 days were used to determine the degradation behavior of the hydrogel, using the following equation:
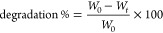
4

### 2.9 Cell Cultivation and Cytotoxicity Measurement by Direct and Indirect Assay

In this study, the NIH3T3 cell line (CRL-1658 TM, ATCC) was used to test the cytotoxicity of HAMA, SFMA, and SFMA–HAMA hydrogels by direct and indirect methods. The NIH3T3 cells were cultured in DMEM (Sigma) with 10% (v/v) fetal calf serum, 100 μg/mL of streptomycin, and 100 μg/mL of penicillin at 37 °C in a humidified 5% CO_2_ atmosphere. HAMA, SFMA, and SFMA–HAMA hydrogels were sterilized for 2 h with 70% ethanol, aspirated, and then allowed to dry overnight in sterile conditions while additionally being irradiated with UV light within a laminar cell culture hood for 30 to 45 min during this procedure.^47,48^ An assessment of biomaterials was conducted in accordance with ISO 10993-5.^49^

To determine indirect contact cytotoxicity, 200 mg of freeze-dried scaffolds were immersed in 1.3 mL of serum-free DMEM for 24 h at 37 °C. The supernatant was then aspirated, and FBS was added to a final concentration of 10%. Subsequently, seeded cells (1 × 10^4^ per well) in 96-well plates were exposed to the extracted medium at dilution factors of 0, 4, and 10. MTT assays were then performed according to the protocol provided by the supplier. To test for cytotoxicity, deionized water was used as a positive control, and cells seeded on tissue culture plates were used as a negative control. The cell viability of each sample was determined by comparing the OD values of the samples to the control.

For the determination of direct contact cytotoxicity, the biomaterials were pre-soaked in DMEM for 2 h under sterile conditions. NIH3T3 cells (at a density of 3 × 10^4^) were seeded into 24-well plates. The biomaterials (4 mm in diameter) were carefully placed at the center of the well and on top of the cells. The hydrogel test samples were incubated for 24 h at 37 °C in an incubator. At the end of the incubation period, the hydrogel test samples were carefully removed with tweezers without contact with the cells, and fresh DMEM/FBS was added. As a negative control, fresh DMEM with 10% FBS without a biomaterial was used. The MTT assay was performed according to the supplier’s protocol. A Hidex plate reader (Hidex Oy, Turku, Finland) was used. The absorbance of the solutions was measured at 570 and 650 nm (as references). By subtracting the absorbances at 570 and 650 nm, the optical density (OD) of the solution was determined.

### 2.10 Live–Dead Viability Assay

For the live–dead assay, cells were seeded in a 96-well plate with 1 × 10^4^ NIH3T3 cells or 5 × 10^4^ MC3T3 cells per well (*n* = 6) and incubated in 100 μL of complete medium (DMEM with 10% FBS for NIH3T3 and αMEM with 10% FBS for MC3T3). After 24 h of incubation, the medium was removed and replaced with 100 μL of material extracts. The cells were then incubated for an additional 24 h. Following 24 h incubation, a live/dead assay was conducted using calcein-AM and propidium iodide according to the manufacturer’s instructions. The cells were treated with full medium as a positive control, while those treated with a 0.1% saponin solution were used as a negative control. For the direct contact test, cells were seeded in a 24-well plate with 1 × 10^5^ NIH3T3 or 5 × 10^5^ MC3T3 cells. After 24 h of incubation in the corresponding medium, a fresh medium was added, 5 mm discs of synthesized materials were placed in wells, and cells were incubated for an additional 24 h. The discs were removed, the medium was replaced with fresh medium, and the live/dead assay was performed according to the manufacturer’s instructions. The results were analyzed using a one-way ANOVA followed by Dunnett’s multiple comparisons test.

Cells were seeded in an Ibidi μ-slide 18-well plate (1 × 10^4^ NIH3T3 cells or 5 × 10^4^ MC3T3 cells per well, *n* = 3) and incubated in 100 mL of full medium (DMEM with 10% FBS for NIH3T3 and αMEM with 10% FBS for MC3T3). After 24 h of incubation, the media were replaced with 100 μL of the corresponding fresh medium, and pieces of the synthesized material covering approximately 25% of the cells were added to each well. After incubation for a further 24 h, materials were removed and replaced with fresh media. Confocal microscopy was performed according to the manufacturer’s protocol.

### 2.11 Statistical Analysis

All experiments were conducted with a minimum of three replicates and results are presented as mean ± SD. The standard *t* test was used for the comparison of statistical significance between the two groups. *p < 0.05, **p < 0.01, and ****p < 0.0001 indicate statistically significant samples.

## 3 Results and Discussion

We schematically depict the synthesis steps of both SFMA (Figure 1A) and HAMA (Figure 1B), which are obtained by reacting SF with GMA and HA with AEMA, respectively. The SFMA reaction involved the nucleophilic addition of the primary amine of SF with GMA through the opening of the epoxide ring, which yielded secondary or tertiary amines containing hydroxyls and methacrylates.^6,27^ The extent of methacrylation was evaluated *via*^1^H NMR spectroscopy (Figure 2A). ^1^H NMR showed an average degree of methacrylation (DoM) for the synthesized SFMA of 41.6 ± 4.5% (Figure 2A,F), which was reproducible across multiple synthesized batches and similar to the previous study done by Kim et al.^6,27^ In this spectrum, peaks located at 5.8 and 6.2 ppm (colored in pink) are attributed to the methacrylate vinyl group protons coming from the MA groups, whereas lysine amino acid peaks are centered at 2.9–3.0 ppm (colored in light green).

The structures of silk cocoon, D-cocoon, SF, and SFMA were examined by ATR-FTIR (Figure 2B). In cocoons and D-cocoons, the peaks of the amide I and II bonds are located at 1620 cm^–1^ and 1525 cm^–1^, respectively, which are shifted to 1636 cm^–1^ and 1525 cm^–1^ in SF.^50^ The peak shifts indicate that the ionic liquid, LiBr solution, disrupts the interaction between polypeptide chains, thereby changing the crystallinity of the SF after extraction and regeneration, as discussed in previous work.^50^ SFMA shows the characteristic peaks at 1640, 1516, and 1240 cm^–1^ for amides I, II, and III, respectively (Figure 2B).

HA modification was achieved *via* the carbodiimide reaction, which is widely used to activate carboxyl groups. HA undergoes most of its chemical modifications in an aqueous solution, where two of its chemical groups, carboxylic and hydroxyl, interact with other components.^51^ To functionalize the carboxyl groups of HA with methacrylate groups using AEMA, we first activated the carboxyl groups with EDC and NHS. The ^1^H NMR spectra of the successfully obtained HAMA also yielded peaks at 5.8 and 6.2 ppm (Figure 2C, pink color). Additionally, a signal from the methyl (−CH_3_) group of AEMA was found at 1.9 ppm (colored in yellow), indicating that AEMA successfully functionalized HA. The synthesized HAMA had shown an average DoM of 32.5 ± 0.7% (Figure 2C,F), which was reproducible for all the synthesis batches. The pristine HA shows an FTIR spectrum at 1602 cm^–1^ and 1411 cm^–1^, which are attributed to COO^–^ group asymmetric and symmetric stretching, respectively (Figure 2D).^31^ Following HA methacrylation, the peak at 1602 cm^–1^ shifts to 1640 cm^–1^ due to an amide bond (amide I) forming between aminoethyl methacrylate and carboxylic acid.^31^ However, the absorption at 1700 cm^–1^ was attributed to the C=O of the AEMA attached to the HA. Furthermore, it is apparent that the intensity of the peaks at 1640 cm^–1^ has increased.^31^ Overall, the FTIR data are consistent with HA modification with AEMA (Figure 2D).

Next, we assessed the molecular makeover using FTIR for the SFMA and SFMA–HAMA composite hydrogels after UV cross-linking (Figure 2E). FTIR spectra of the SFMA–HAMA composites revealed absorption bands that are distinctive to each component: SF, HA, SFMA, and HAMA (Figure 2B,D). The two bands located at 1230 cm^–1^ (corresponding to SF and SFMA) and 1045 cm^–1^ (representing HA and HAMA) exhibit distinct separation from the other bands, allowing the identification of these components (Figure 2E). The intensity of the band at 1045 cm^–1^ increased in the SFMA–HAMA hydrogels with the incorporation of HAMA into the matrix as compared to the pristine SFMA (5, 10, and 20 wt %).

The obtained Fourier transform spectra self-deconvolution (FSD) of the amide I region (1600–1700 cm^–1^) was performed to quantify the crystallinity level in order to comprehend the impact of increasing SFMA concentrations and incorporating HAMA1 into the SFMA network (Figure S1). Although no significant differences were observed in the random coil content among the six groups, noteworthy variations were evident in the β-sheet, β-turn, and α-helix contents. It can be observed that when the concentration increases, the β-sheet content increases as well; specifically, the values for SFMA5, SFMA10, and SFMA20 were approximately 62.8 ± 0.1, 62.1 ± 5.3, and 73.3 ± 5.7%, respectively. Furthermore, the β-turn content slightly increased from 18.3 ± 1.0% in SFMA5 to 19.7 ± 4.5% in SFMA20, while the α-helix content decreased from 18.8 ± 1.1% in SFMA5 to 1.9% ± 1.1 in SFMA20. After adding HAMA into SFMA5 and SFMA10, the content of the β-sheet did not change much and was in the same range; however, the content of the β-sheet increased from 73.3 ± 5.7 to 86.9 ± 9.3% by adding HAMA1 into SFMA20 networks (Figure S1). Here, we acknowledge the difficulty of the exact analysis of the removal of the HAMA contribution to the composite sample deconvolution, which may partly influence the overall results.

**Figure 3:**
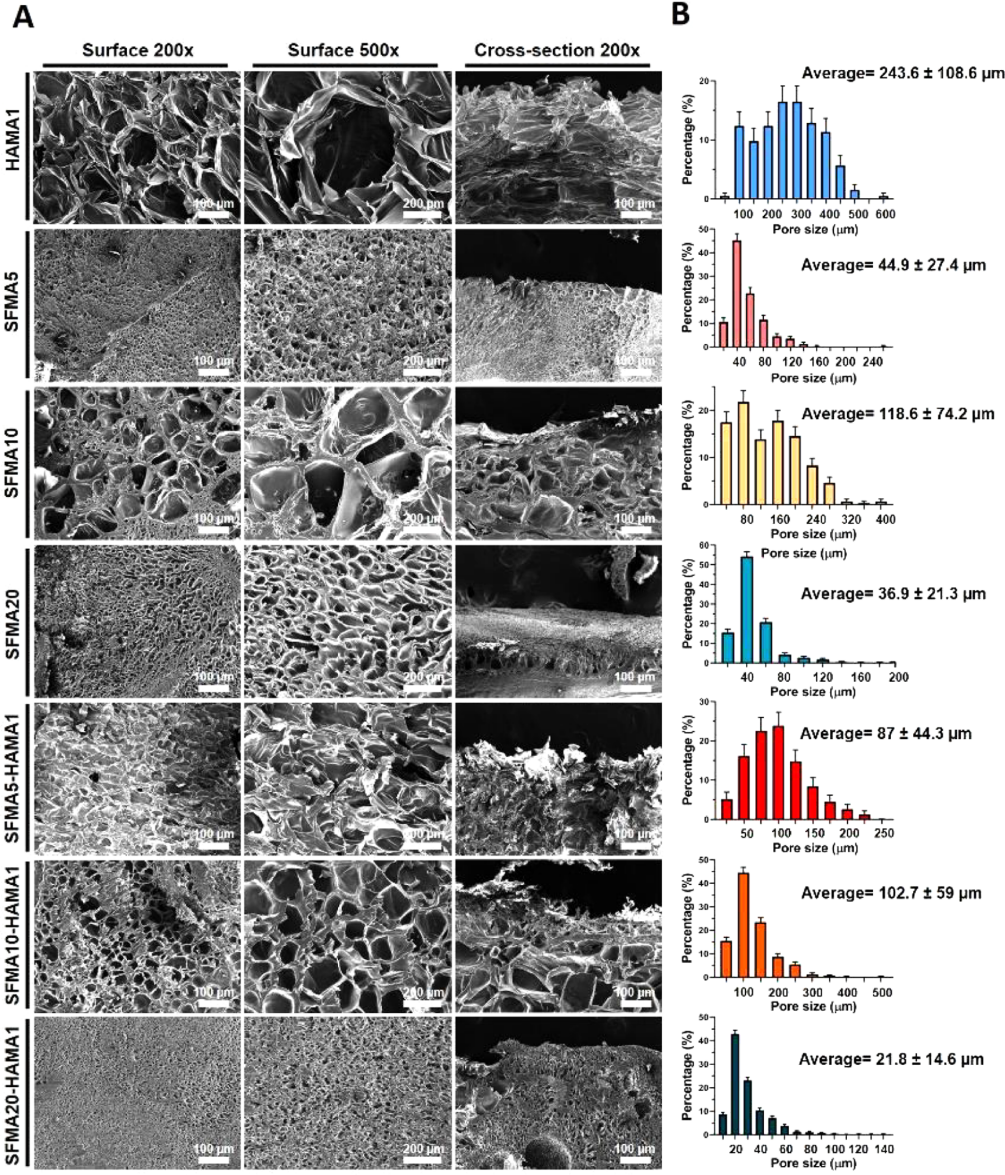
SEM images of (A) HAMA, SFMA, and SFMA–HAMA hydrogels after UV (365 nm) curation in the presence of 0.4 wt % LAP and (B) pore size distribution within the imaged hydrogels were analyzed with ImageJ and plotted. Log-normal distributions were plotted through each histogram in panel B and the averages from it are noted in the graph.

The SEM results shown in Figure 3A indicate that all hydrogels after lyophilization had porous structures both on the surface and in cross-section. Image analysis was carried out on HAMA, SFMA, and SFMA–HAMA lyophilized hydrogels using ImageJ software to determine the average diameter of the pores. The overall trend demonstrates that the pore size decreased as the concentration of SFMA increased from 5 to 20 wt %. However, SFMA10 displayed a larger pore size than SFMA5 and SFMA20. The porosity observed in SEM describes the morphology of hydrogels after freeze-drying, and it should not be regarded as descriptive of the gels in the physiological hydrated state. However, morphology analysis from SEM can reveal the relevant features of these composites. The increased pore diameter in SFMA10 could be explained by the increased wall thickness compared with that of SFMA5. By increasing the wall thickness, the pore density decreases, resulting in an increase in average pore diameters per unit volume.^52,53^ Increasing the concentration of SFMA to 20% led to a simultaneous reduction in the size of the pores and an increase in the thickness of the walls. By incorporating HAMA into the SFMA10 and SFMA20 matrixes, the pore size could be reduced over their pristine states without HAMA. As shown in Figure 3B for all composite samples, the SFMA5–HAMA1, SFMA10–HAMA1, and SFMA20–HAMA1 ranges overlap with those without HAMA1. Albeit there are some differences in shifts of the distributions, the overall addition of HAMA to the SFMA network does not seem to impair the overall morphology.

**Figure 4:**
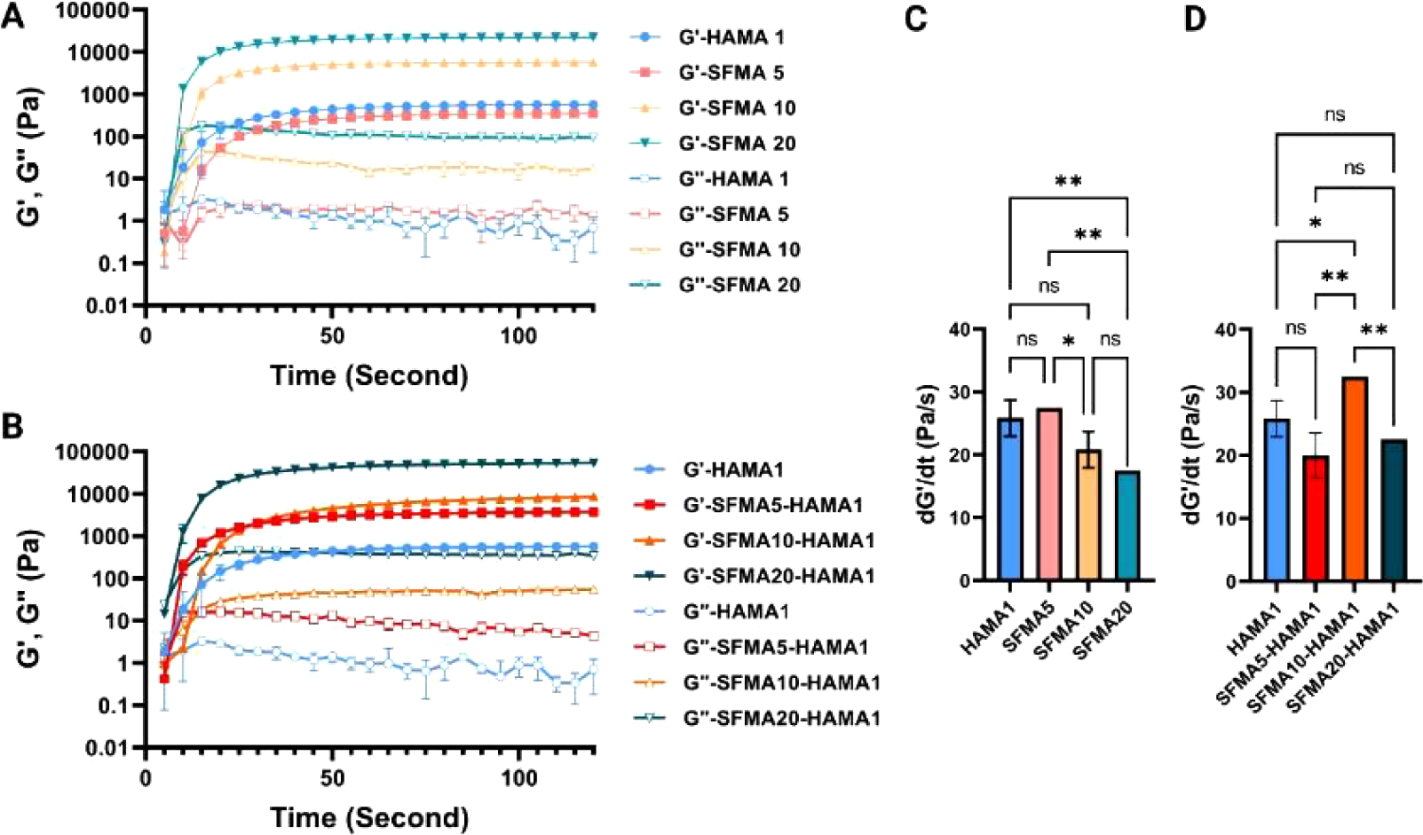
Gelation kinetics of (A) HAMA1, SFMA5, SFMA10, and SFMA20; (B) HAMA1, SFMA5–HAMA1, SFMA10–HAMA1, and SFMA20–HAMA1; (C) gelation time for HAMA1, SFMA5, SFMA10, and SFMA20; and (D) gelation time for HAMA1, SFMA5–HAMA1, SFMA10–HAMA1, and SFMA20–HAMA1 hydrogels at 37 °C (*n* = 3 represents three measurements taken from the same batch of samples; the bars are smaller than data marks). The *in situ* gelation process was measured on the quartz crystal stage by illuminating the bottom of the deposited sample with UV light. Data represented as the mean and standard deviation of *n* = 3 repeats (ns = not significant, *p < 0.05, **p < 0.01).

Rheology was used to investigate the viscoelastic properties of HAMA, SFMA, and SFMA–HAMA hydrogels. First, we investigated the gelation kinetics of the synthesized SFMA (at 5, 10, and 20 wt %) and HAMA (1 wt %) under the influence of UV irradiation (Figure 4A,C), as well as their SFMA–HAMA composites (Figure 4B,D). Initially, the loss moduli (*G*″) in all samples were higher than their storage moduli (*G*′), confirming that all samples behaved as solutions. Upon UV irradiation, which was switched on 10 s after starting the rheological measurement, *G*′ increased as expected, rapidly surpassing the *G*″ and indicating transition from a solution to a soft-solid, hydrogel state. In each case, *G*′ was increasing until it reached plateau, determined by the availability of the LAP photoinititator and the number of mechanically active cross-links achievable for each network. We noticed that HAMA with 1 wt % and SFMA with 5 wt % carried similar plateau storage moduli (Figure 4A) in the ranges 517 ± 22 and 313 ± 4 Pa, respectively. Next, the increase in SFMA concentration led to 18.9-fold and 73.7-fold increases in storage moduli for 10 and 20 wt %, respectively (Figure 4A). Gelation times for all samples are summarized in Figure 4C,D. HAMA 1 wt % and SFMA 5 wt % exhibited similar gelation times of 25.8 ± 2.9 and 27.5 ± 0.0 s (repeats gave identical values), respectively (Figure 4C). The increase in the SFMA concentration was associated with shortened gelation time, decreasing to about 20.8 ± 2.9 and 17.5 ± 0.0 s (repeats gave identical values) for 10 and 20 wt % SFMA, respectively. While for 5 and 20 wt % SFMA, addition of HAMA 1 wt % resulted in higher stiffness, for 10 wt % SFMA–HAMA, similar storage modulus was retained (Figure 4B). It should also be noted that SFMA–HAMA composites exhibit different gelation trends, ranging from 20.0 ± 3.5 s to 22.5 ± 0.0 s (repeats gave identical values) for SFMA5–HAMA1 and SFMA20–HAMA1 (Figure 4D). Clearly compositing SFMA with HAMA shifts the dominant concentration effects from SFMA due to the secondary interactions between the two polymers. Hence, we investigated further these effects by additional rheological characterization below.

**Figure 5:**
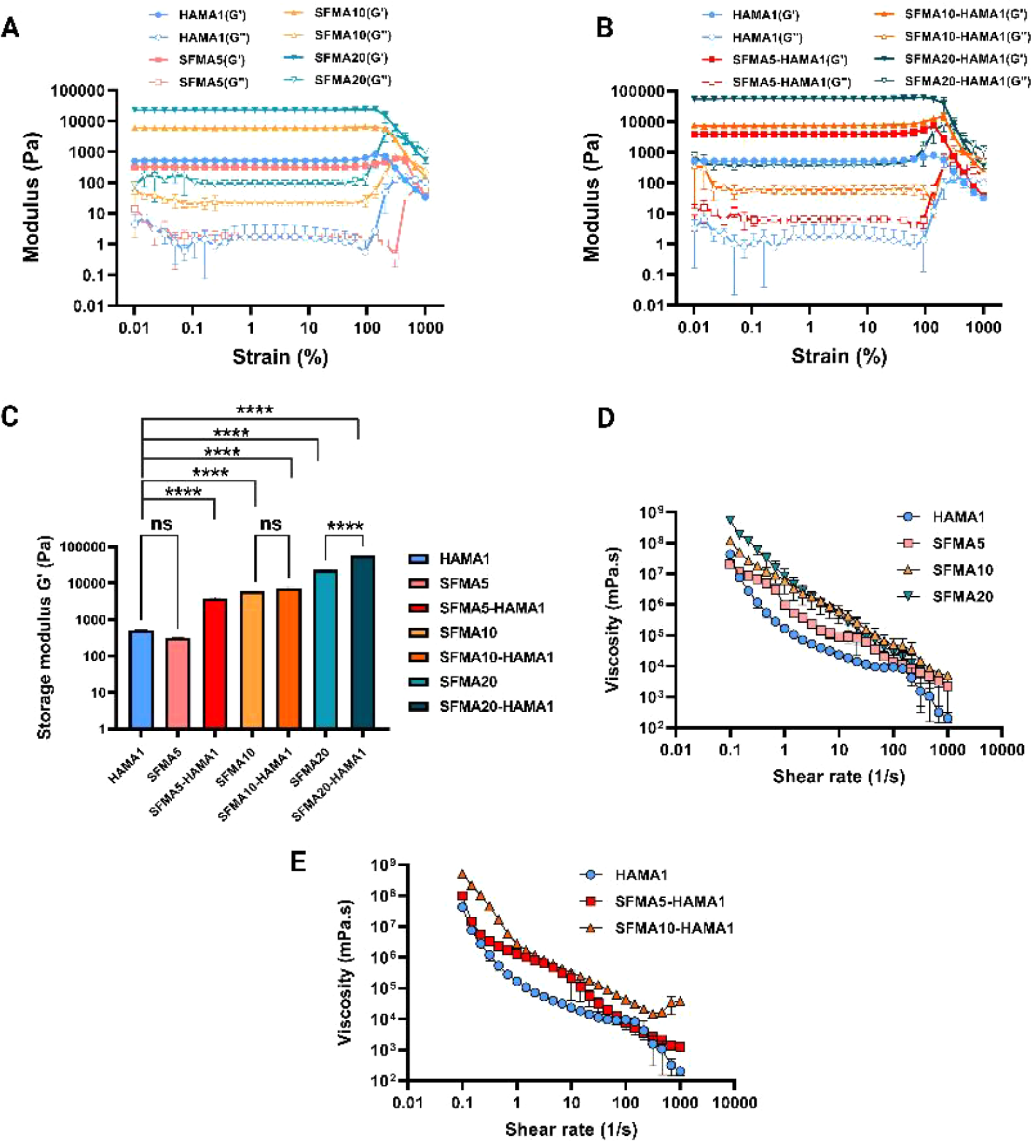
Hydrogel rheological characterization. (A) Amplitude sweep of HAMA1 and SFMA5, SFMA10, and SFMA20. (B) Amplitude sweep of HAMA1, SFMA5–HAMA1, SFMA10–HAMA1, and SFMA20–HAMA1. (C) Storage modulus (mechanical stiffness) of HAMA1, SFMA (5, 10, and 20 wt %), SFMA5–HAMA1, SFMA10–HAMA1, and SFMA20–HAMA1 hydrogels obtained from amplitude sweeps at 1 Hz and 0.2%. (D) Viscosity curves for HAMA1 and SFMA (5, 10, and 20 wt %) hydrogels. (E) HAMA and SFMA5–HAMA1, SFMA10–HAMA1, and SFMA20–HAMA1 hydrogels. Data represented at the mean and standard deviation of *n* = 3 repeats (ns = not significant, ****p < 0.0001).

All control and composite UV-cross-linked hydrogels were initially subjected to an amplitude sweep (Figure 5A,B). In all cases, storage shear moduli (*G*′) were observed to be an order of magnitude larger than the loss moduli (*G*″), confirming the typically solid-like nature of these hydrogels. Up until a strain of ≈100% (Figure 5A,B), all hydrogels (HAMA, SFMA, and SFMA–HAMA) displayed a clearly defined linear viscoelastic region (LVR). The addition of HAMA to SFMA did not result in a significant shift of the crossover points (*G*′ = *G*″) for higher SFMA concentrations, with the average crossover points at 772 ± 0.25% strain and 739 ± 10% obtained for SFMA10 and SFMA20, respectively, and 766 ± 14% strain and 734 ± 10% for SFMA10–HAMA1 and SFMA20–HAMA1, respectively. The only exception to this was the crossover point for the SFMA5 sample, which decreased from 971 ± 27% to 731 ± 4% strain upon compositing with HAMA, indicating the decrease of composite’s resilience to strain and dominance of more viscous HAMA in the overall viscoelastic behavior.

The *G*′ and *G*″ values of the SFMA and SFMA–HAMA hydrogels increased with increasing concentrations of SFMA (Figure 5A–C). As already observed, both HAMA1 and SFMA5 carried a similar storage modulus (Figure 5C). Upon increasing the SFMA concentration from 5 to 10 and 20 wt %, *G*′ increased from 313 ± 4 to 5910 ± 170 Pa and 23 070 ± 620 Pa, respectively (Figure 5C). For the SFMA5–HAMA1 and SFMA20–HAMA1 composite hydrogels, the overall mechanical stiffness (storage modulus *G*′) increased by the addition of HAMA to the SFMA networks, resulting from the increased total polymer content (Figure 5C,D). In contrast, the mechanical stiffness of the SFMA10–HAMA1 was similar to that of SFMA10 (Figure 5C,D), as is related to the similar sample topology as evidenced by similar pore size of the SFMA10–HAMA1. The mechanical stiffness of hydrogel is affected by a variety of parameters, including the degree of functionalization, porosity, wall thickness, UV intensity, and concentration.^54^ Considering that the methacrylation degree and UV intensity were the same in all groups of samples, mesh size and wall thickness are the variables that determine the stiffness of the sample.^55^

For extrusion-based 3D bioprinting or injectability of formulations, another characteristic that must be considered is the viscosity of the hydrogel precursor. Schwab et al.^56^ recently highlighted that for 3D-extrusion-based techniques, the inks usually start from a resting state and undergo a structural transition due to high shear while passing through the nozzle. Then, they re-establish the original resting state on deposition, with both processes governed by the key rheological characteristics, including shear thinning and shear recovery.^57^ Here, printability/injectability of the developed inks was initially evaluated based on shear-thinning behavior of the inks precross-linked for 2 min (Figure 5D,E). The SFMA and SFMA–HAMA hydrogel precursors’ viscosities were tested between 0.1 and 1000 s^–1^ of shear rate, and the resulting flow curves are shown in Figure 5D,E. The viscosity of the SFMA hydrogel precursors rose in a manner similar to how *G*′ elevated as a function of SFMA concentration. Figure 5D demonstrates that all hydrogel precursors, including HAMA and SFMA, had viscosities that gradually decreased as shear rates increased, indicating a flow behavior under high shear that made the hydrogel precursors appropriate for extrusion. For SFMA–HAMA, however, the trend was quite different. After incorporating the HAMA into the SFMA with a concentration of 5 wt %, the viscosity decreased steadily, whereas SFMA10–HAMA1 followed trends that were completely distinct from those of SFMA5–HAMA1. As illustrated in Figure 5E, the viscosity of SFMA10–HAMA1 initially decreased with the increasing shear rate; however, it began to increase above a shear rate of 681 1/s. In the SFMA10–HAMA1 hydrogel, three flow behaviors were observed with increasing shear rates. The first was rapid shear thinning followed by moderate shear thinning, while at higher shear rates, above 100 1/s, shear thickening was observed. The viscosity of the SFMA10–HAM1 rapidly decreased from 0.1 to 1 1/s and moderately decreased from 1 to 100 1/s due to the disentanglement of molecules or chain alignment under the shear force. However, when the shear rate was further increased to more than 100 1/s, the SF began to dehydrate (shear-induced phase separation)^58^ and underwent conformational changes,^59^ and this led to an increase in viscosity. On the basis of previous studies by Zhang and co-workers, the shear thickening of the regenerated SF solution has been attributed to the formation of intermolecular interactions during the full extension of the SF chains at high shear rates.^58,60^ In addition, the formation of β-sheet structures at higher shear rates may lead to accelerated solidification based on the Boulet-Audet et al.’s study.^58,61^ This is one of the challenges in printing SF-based materials, as they undergo conformational changes under shear stress.^59^ To overcome this, Costa et al. suggest using a lower concentration of SF in formulating the bioink.^59^ The flow curve of SFMA20–HAMA1 was not measurable due to the viscosity that was too large (nearer to solid state) of this sample.

**Figure 6:**
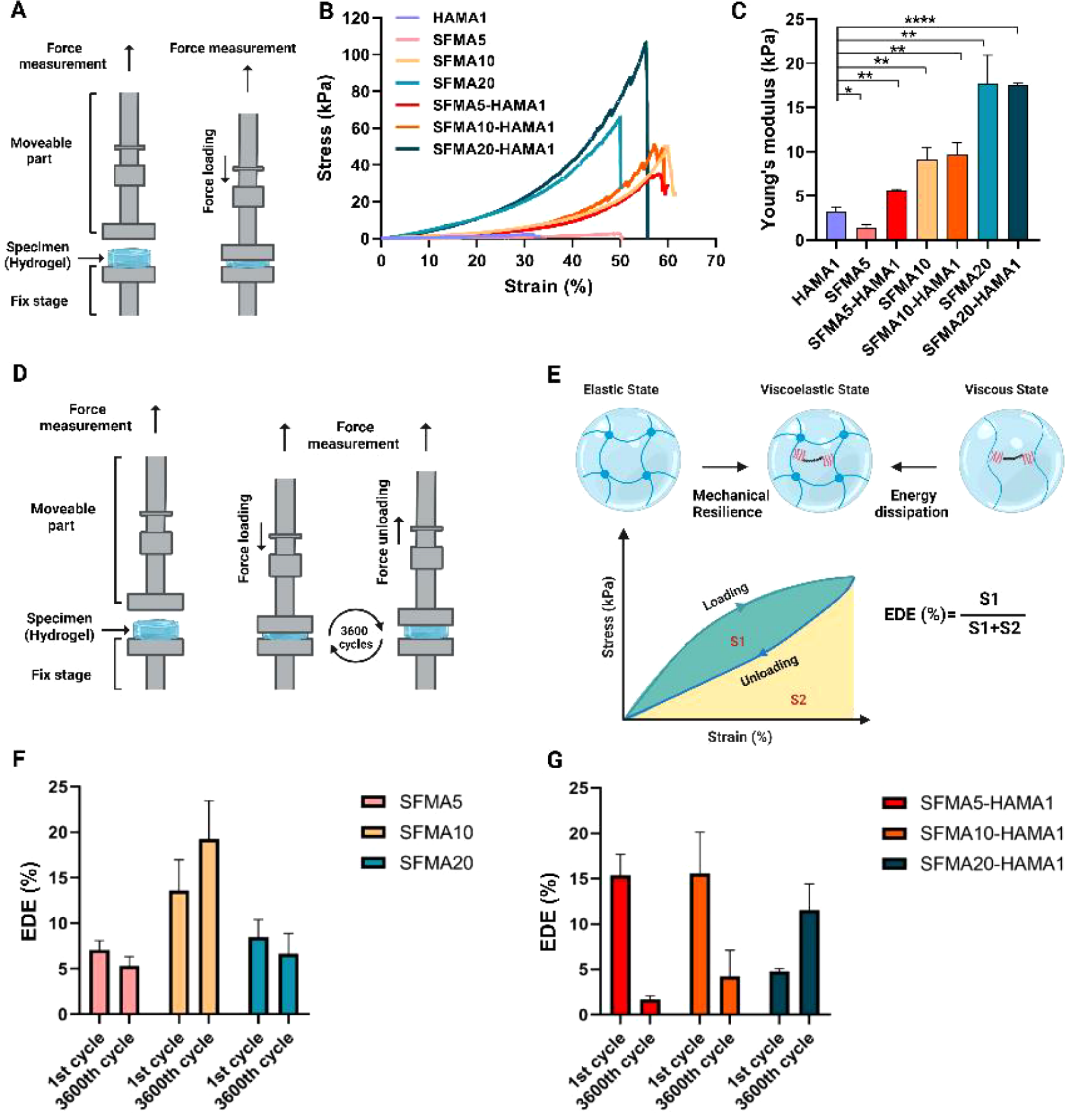
Measurements of mechanical properties under static and dynamic conditions. (A) The illustration and working principle of the static mechanical analysis; (B) compressive strength measurement of the HAMA1, SFMA5, SFMA10, SFMA20, SFMA5–HAMA1, SFMA10–HAMA1, and SFMA20–HAMA1 hydrogels in static condition; (C) obtained Young’s modulus values from static mechanical measurements for all formulations. (D) the illustration and working principle of the dynamic mechanical analysis; (E) the representation of the schematic for calculating energy dissipation efficiency (EDE) (%); (F) measure value of EDE (%) SFMA5, SFMA10, and SFMA20; and (G) measure value of EDE (%) SFMA5–HAMA1, SFMA10–HAMA1, and SFMA20–HAMA1. Parts A and D and the scheme in panel E were created with Biorender.com. Data represented at the mean and standard deviation of *n* = 3 repeats. *p < 0.05, **p < 0.01, ****p < 0.0001.

The mechanical and viscoelastic properties of tissue-engineered hydrogels play a significant role in the guidance of cellular behavior;^62^ hence, we measured the bulk mechanical properties of the obtained hydrogels. Figure 6A presents a schematic overview of the procedure for measuring the static compressive strength. With respect to the compressive strength test, HAMA1 had the lowest stress value of 2.29 ± 0.57 kPa and the lowest strain value of 29.5 ± 6.5 kPa (Figure 6B and Table 2). For SFMA, as the concentration increased from 5% to 10% and 20%, the corresponding stress values were 2.71 ± 0.40, 50.0 ± 0.9, and 66.0 ± 23.9 kPa, respectively. The strain values for these concentrations were 50 ± 2.00, 60.1 ± 0.02, and 50.0 ± 0.02%, respectively. Incorporating the HAMA1 into the SFMA5 and SFMA20 networks results in improved stress values of 24.7 ± 18 and 89.5 ± 14.7 kPa, respectively, which are almost 9.1 and 1.4 times higher than the values for SFMA5 and SFMA20. The stress values for SFMA10 and SFMA10–HAMA1 were in a similar range, measuring 50.0 ± 0.9 and 35.66 ± 18.3 kPa, respectively. Table 2 provides a detailed breakdown of the data for the measurements and comparisons discussed, allowing further analysis and comparison of the results to other relevant literature. Based on Figure 6C, it is evident that adding HAMA1 to SFMA networks significantly increases the material’s stiffness, as indicated by the values of Young’s modulus. HAMA1 and SFMA5 have low stiffness on their own, but as SFMA concentration increases (from SFMA5 to SFMA20), stiffness also increases. A substantial increase in Young’s modulus occurs when HAMA1 is combined with SFMA, particularly in the SFMA20–HAMA1 network, which produces the highest Young’s modulus. Thus, increasing SFMA concentrations and adding HAMA1 both enhance mechanical strength.

**Table 2:** Comparison of our Results with Other References, Detailing Parameters Like Concentration, Degree of Methacrylation, Swelling Ratio, Swelling at 5 h, Compressive Strength (kPa), Compressive Strain (%), Storage and Loss Modulus at 0.2%, and the Condition of Measurement (Gel or Dried)^a^

Samples name		LAP or IQ (w/v)	Degree of methacrylation (DoM) %	Swelling ratio	Swelling at 5 h	Compressive strength (kPa)	Compressive strain (%)	Storage modulus (Pa) at 0.2%	Loss modulus (Pa)	Condition	ref
SFMA15%	pH 5	0.5% LAP	35–40	39.3 ± 3.7	NM	40 ± 4	NM	3600±400	NM	gel	(37)
SFMA15%	pH 7	0.5% LAP	35–40	80.9 ± 11.9	NM	21 ± 2	NM	900±100	NM		
SFMA15%	pH 8	0.5% LAP	35–40	86.2 ± 7.9	NM	18 ± 1	NM	500±50	NM		
SFMA10%–GMA 424 mM		0.2% LAP	42	NM	4580 ± 480	122 ± 45	69.5 ± 0.5	100	10	gel	(27)
SFMA20% 424 mM		0.2% LAP	42	NM	2026 ± 202	434 ± 128	77.7 ± 3.3	900	120	gel	(27)
SFMA25%		0.3% LAP	42	NM	NM	636	72.6	∼500	30	gel	(28)
SFMA30% 424 mM		0.2% LAP	42	NM	1059 ± 121	910 ± 127	80 ± 5.1	2750	425	gel	(27)
SFMA-L		0.1% IQ	44.5	NM	1200	5.4 ± 0.3	NM	200–300	55	gel	(59)
SFMA-M		0.1% IQ	57.2	NM	800–900	11.6 ± 1.2	NM	200–300	55	gel	(59)
SFMA-H		0.1% IQ	63.5	NM	600–700	24.4 ± 2.8	NM	200–300	40	gel	(59)
SFMA20%-GMA 176 mM		0.3% LAP	NM	380–400	300	550	65	NM	NM	gel	(76)
SFMA10%-chemical cross-linked 10GMA in 100 mL		1% LAP	NM	NM	NM	7000	17–20	NM	NM	dried sponge	(33)
SFMA10%-physical cross-linked 10GMA in 100 mL		1% LAP	NM	NM	NM	2700–2800	65–68	NM	NM	dried sponge	(33)
HAMA1		0.4% LAP	32.5 ± 0.7	NM	NM	2.29 ± 0.57	29.5 ± 6.5	517 ± 22	1.4 ± 1.4	gel	current work
SFMA5		0.4% LAP	41.6 ± 4.5	66.2 ± 2.4	64.8 ± 5.1	2.71 ± 0.40	50 ± 2.00	313 ± 4	2 ± 0.6	gel	current work
SFMA10		0.4% LAP	41.6 ± 4.5	258 ± 50	228 ± 27	50.0 ± 0.9	60.1 ± 0.02	5910 ± 170	23 ± 9.2	gel	current work
SFMA20		0.4% LAP	41.6 ± 4.5	167 ± 36	173±21	66.0 ± 23.9	50.0 ± 0.02	23070 ± 620	93 ± 2.4	gel	current work
SFMA5–HAMA1		0.4% LAP	41.6 ± 4.5	1091 ± 173	1058± 188	24.7 ± 18	50.3 ± 9.5	3850 ± 244	5.9 ± 1	gel	current work
SFMA10–HAMA1		0.4% LAP	41.6 ± 4.5	345 ± 78	341 ± 68	35.66 ± 18.3	54.5 ± 6.4	7319 ± 531	61 ± 14	gel	current work
SFMA20–HAMA1		0.4% LAP	41.6 ± 4.5	122 ± 36	117 ± 29	89.5 ± 14.7	51.8 ± 3.1	56624 ± 1045	378 ± 35	gel	current work

a NM: not mentioned, IQ: Irgacure 2959.

To further ascertain the mechanical properties of our hydrogels, we assessed their stress–strain curves from 0 to 3600 cycles with and without cyclic loading (Figure S2). Figure 6D depicts the experimental setup utilized to assess the mechanical characteristics of hydrogels under cyclic loading. During the course of the experiment, the hydrogels were situated between stationary and mobile components. They were subjected to loading and unloading over 3600 cycles, with force and displacement recorded at regular intervals throughout. This study used a similar protocol for cyclic recovery to the cartilage tissue experiments,^63^ providing an applicable comparison for the evaluation of the durability of the hydrogels. These analyses enabled the calculation of several key parameters, including compressive modulus, durability, material toughness, and amount of dissipated energy.

One of the parameters we evaluated was the hysteresis during loading and unloading from 0 to 3600 cycles. Hysteresis is a term used to describe the time-dependent dissipation of thermal energy by viscoelastic materials. The assessment is conducted through a comparison between the differences in the areas under the stress–strain curves, which are plotted for both the loading and unloading of the specimens. One way to look at this is by calculating the energy dissipation efficiency factor (EDE%). The illustration in Figure 6E shows this concept of EDE% when mechanical testing is performed. Stress and strain are shown during the loading and unloading phases. We calculated the EDE% based on the ratio between the area under the loading curve (S1) and the total area under both the loading and unloading curves (S1 + S2). This indicates the material’s degree of mechanical resilience and its ability to dissipate energy. Figure 6F shows the representative EDE% for the SFMA5, SFMA10, and SFMA20 hydrogels during the first and 3600th cycles. SFMA10 exhibited an increase in EDE% at 3600 cycles compared to the first cycle, whereas SFMA5 and SFMA20 displayed a decrease in EDE% at 3600 cycles compared to the first cycle. Furthermore, we also assessed the EDE% after introducing HAMA1 into SFMA5, SFMA10, and SFMA20 hydrogels. The EDE% for SFMA5–HAMA1, SFMA10–HAMA1, and SFMA20–HAMA1 hydrogels during the first and 3600th cycles is shown in Figure 6G. As shown in Figure 6G, incorporating HAMA1 into SFMA5 resulted in a decrease in EDE% at 3600 cycles compared to the first cycle. We observed the same trend for SFMA10–HAMA1. However, SFMA20–HAMA1 exhibits a different trend from SFMA5–HAMA1 and SFMA10–HAMA1, and EDE% increases at 3600 cycles compared to the first cycle.

Additionally, we have considered the first, 900th, 1800th, 2700th, and 3600th cycles in order to reduce the total amount of data (Figures 6D,E and S2) in hysteresis loops. Hysteresis energy is one of the parameters measured in this study, which depends on a number of variables, including the applied load itself, any previous loads that may have been applied, and the rate at which the current load is applied. During the process of loading, a material frequently undergoes internal friction as molecules slide past one another. This phenomenon generates heat, which subsequently dissipates into the surrounding environment. Figure S2A shows the stress curve for the hydrogel at 1, 900, 1800, 2700, and 3600 cycles of loading and unloading. Data reveal that the hysteresis hoop exhibited good elasticity during loading and unloading cycle tests conducted on SFMA (5, 10, and 20), as well as SFMA5–HAMA1, SFMA10–HAMA1, and SFMA20–HAMA1. Figure S2A shows that the first hysteresis loops of SFMA5, SFMA20, SFMA5–HAMA1, and SFMA10–HAMA1 had relatively large areas, but the areas of their 900th, 1800th, 2700th, and 3600th loops became relatively small and were highly consistent with each other. Nevertheless, the initial hysteresis loop for SFMA10 and SFMA20–HAMA1 was relatively small, as seen in Figure S2A. Conversely, the 900th, 1800th, 2700th, and 3600th hysteresis loops had relatively high areas. As stated in the existing literature, an increase in the peak stress during cyclic loading results in hardening of the material; conversely, a decrease leads to a softening. The peak stress in all groups of hydrogels slightly decreases.

Additionally, we have included the stress and strain data for the first and last 5 cycles, 0–5 cycles, and 3595–3600 cycles, respectively, as shown in Figure S2B, to demonstrate the stability of these values at both the beginning and the end of the 3600 cycles. The increase in the concentration of SFMA resulted in an increase in the force of SFMA20 hydrogel compared to SFMA20, which is in agreement with previous studies (Figure S2C1–C3).^64^ Furthermore, the stability of the force amplitude versus cycles in SFMA5, SFMA10, and SFMA20 hydrogel shows that the force initially is high and the end of the 3600 cycle slightly decreases (Figure S2C1–C3). This slight decrease could be due to minor structural relaxation or degradation of SF. It may be caused by microcracks forming within β-sheet regions in the hydrogel network that gradually reduce the force needed to deform the gel. However, after adding the HAMA1 to the SFMA5, SFMA10, and SFMA20 networks, the initial force is low. As the number of cycles increases, the force increases (Figure S2C4–C6). Figure S1E presents the dynamic mechanical stiffness of SFMA (5, 10, and 20 wt %) and SFMA–HAMA hydrogel samples. As illustrated in Figure S2E1–E3, the dynamic stiffness of SFMA5, SFMA10, and SFMA20 exhibits a decline with the progression of loading cycles. However, following the incorporation of HAMA1 into SFMA5, SFMA10, and SFMA20, the initial stiffness was observed to be relatively low but demonstrated an upward trend over time, slightly decreased at the end of the testing cycles (Figure S2E4–E6). Therefore, SFMA–HAMA hydrogel properties can be tailored for a wide range of applications in biomedical research and related fields including tissue engineering and regeneration.^63,65^ The other hydrogels, SFMA20 and SFMA20–HAMA1, contain higher concentrations of SFMA, which could make them potentially suitable for testing cartilage tissue engineering applications. SFMA10 and SFMA10–HAMA1 hydrogels, along with SFMA5–HAMA1 hydrogels, possess an intermediate stiffness that enables their application in tissues that necessitate a balance between elasticity and rigidity, such as connective tissue.

**Figure 7:**
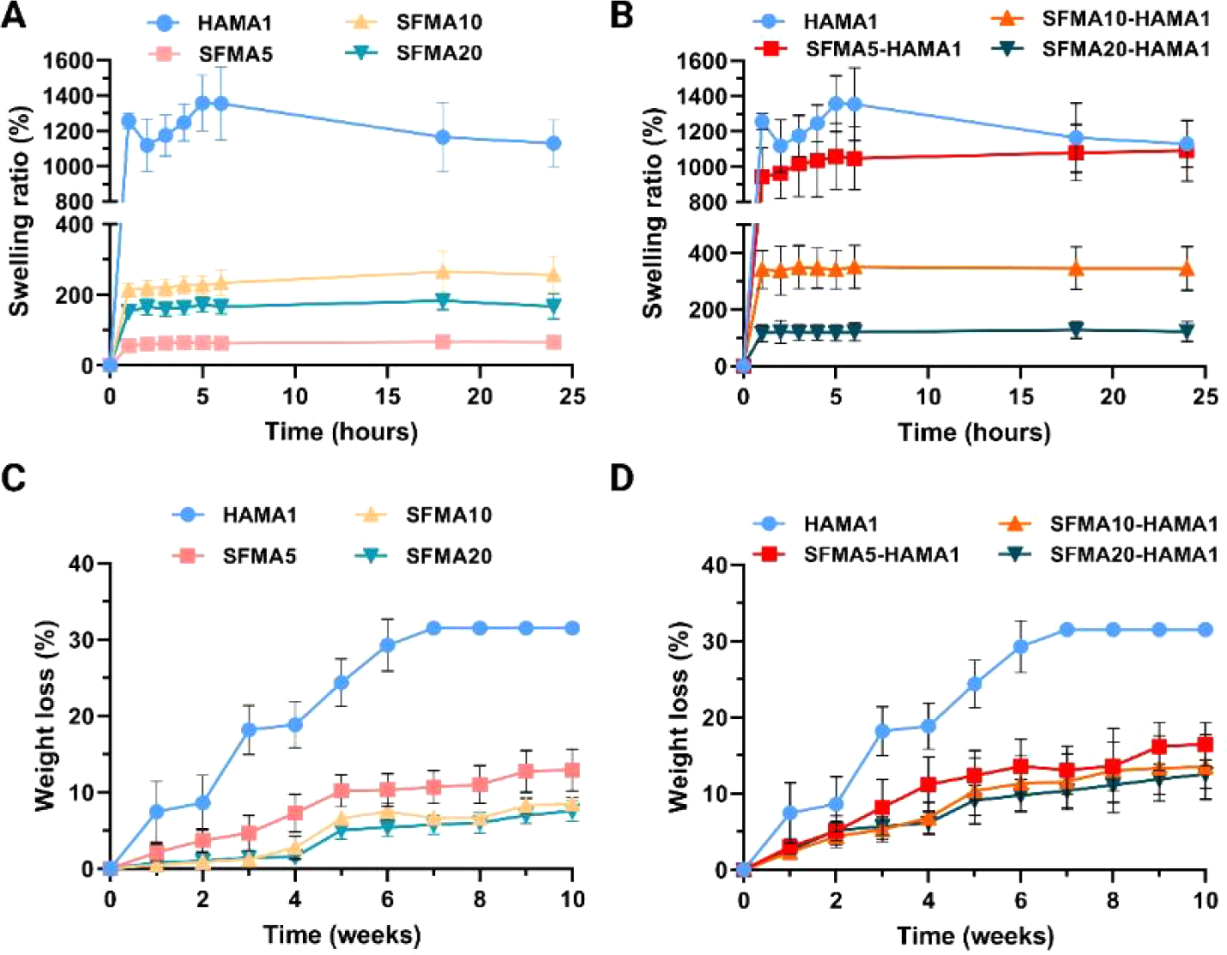
Swelling ratio of (A) HAMA1 and SFMA (5, 10, and 20%) and (B) HAMA1, SFMA5–HAMA1, SFMA10–HAMA1, and SFMA20–HAMA1 hydrogels and degradation percent of the (C) HAMA1 and SFMA (5, 10, and 20%) and (D) HAMA1, SFMA5–HAMA1, SFMA10–HAMA1, and SFMA20–HAMA1 hydrogels. Data shown in the mean (*n* = 3) and SD are shown. The bars are smaller than data marks.

The swelling behavior of the hydrogel is a key factor in determining how effectively the components produced will adhere to the surrounding tissue.^27^ All hydrogel groups, including HAMA, SFMA, and SFMA–HAMA hydrogels, underwent 24 h uptake tests in PBS. In the first hour (1 h) of the experiment, SFMA5, SFMA10, and SFMA20 displayed swelling values of 55 ± 2, 214 ± 18, and 153 ± 15%, respectively (Figure 7A). First, we note that SFMA5 has the lowest swelling compared to SFMA10 and SFMA20. This correlates very well with the condensed structure for this hydrogel and subsequently its highest degradation rate (Figure 7C), which is in agreement with previous research.^1^ However, with regard to swelling and degradation rates, a key point to keep in mind is that these parameters, while related, do not necessarily correlate linearly. A hydrogel’s swelling ratio can be used as an indicator of its ability to absorb water, which is influenced by both the cross-linking density and the hydrophilic nature of the polymer network.^66^ The swelling ratio of a material is typically higher when it is less densely cross-linked and has a higher porosity, allowing for increased water absorption. In contrast, the degradation rate is affected by both the hydrogel’s chemical structure and its cross-linking density, as well as the environmental conditions at the time of degradation. Increasing the cross-linking density produces a denser network, which degrades more slowly due to its increased stability and reduced susceptibility to hydrolysis or enzymatic breakdown.

Prior research shows that increasing the concentration of SF accelerates its transformation from a random coil and α-helix structure to a β-sheet structure, which helps to create more β-sheet structures in SF.^67^ The investigation conducted by Kambe et al. demonstrated a link between the β-sheet concentration and the degradation time of the SF.^68^ Increasing the fraction of β-sheets reduces SF’s enzymatic and hydrolytic degradation while increasing the scaffold stability and degradation time.^69,70^ SFMA5 and SFMA10 have lower β-sheet content than SFMA20, resulting in a higher degradation rate (Figure S1).

As the concentration of the SFMA increased from 10 to 20 wt %, swelling diminished. According to these results, the SFMA10 hydrogel was demonstrated to absorb water at a rate 1.5 times greater than that of the SFMA20 hydrogel and 4 times greater than that of the SFMA5 hydrogel. Following 24 h of immersion in PBS, the swelling behavior of the SFMA5, SFMA10, and SFMA20 was 66 ± 2, 258 ± 50, and 167 ± 36%, respectively. Numerous factors influence the swelling behavior of the hydrogels, including hydrophilic groups, cross-linking density, chain flexibility, free volume, and electrostatic attraction.^1^ The materials are identical, differing only in their concentration, which determines the pore size, a critical factor influencing swelling behavior (Figure 7A).

Upon hydration for HAMA1 hydrogels in the first hour, the swelling was 1256 ± 46%, but after 6 h, water uptake increased to 1357 ± 208%, and thereafter, the HAMA1 hydrogels reached a steady state (Figure 7A,B). In contrast, SFMA–HAMA hydrogels primarily display a HAMA1 hydrogel water uptake trend, indicating that HAMA molecules are predominating the trend by directly binding water molecules to its structure. During the initial hour of the investigation, the swelling results for SFMA5–HAMA1, SFMA10–HAMA1, and SFMA20–HAMA1 were as follows: 940 ± 170, 341 ± 68, and 116 ± 30%, respectively. The swelling behavior decreased with an increase in the concentration of SFMA when the HAMA1 is added to it at three distinct concentrations. With respect to the other two SFMA–HAMA composite hydrogels, SFMA5–HAMA1 exhibits the highest swelling behavior, which demonstrates the domination of HAMA characteristics in this composite hydrogel. SFMA5–HAMA1, SFMA10–HAMA1, and SFMA20–HAMA1 exhibited swelling ratios of 1091 ± 173, 345 ± 78, and 122 ± 36%, respectively, after 24 h of immersion in PBS (Figure 7B).

Biomaterial degradation behavior plays an important role in tissue engineering. It can also facilitate the control of the release rate of the therapeutic agent and improve its efficacy in tissue engineering.^1^ There are various biological, chemical, and physical processes, which can influence biodegradation rates.^71^ These processes are influenced by the characteristics of the polymer and the local surrounding environment (pH, salt, and enzyme concentration and types of cells around) within the body.^71^ These can be simulated in *in vitro* tests to get the overall predictions before any planned *in vivo* testing. Figure 7C,D shows the hydrogel degradation properties of SFMA, HAMA, and SFMA–HAMA hydrogels when placed in PBS. Initially, we evaluated the impact of increasing the SFMA concentration from 5 to 20 wt % on the degradation process. The results in Figure 7C indicated that the degradation ratio decreased with increasing concentration of SFMA from 5 to 10 and 20 wt %. After 5 weeks, the amount of degradation for SFMA5, SFMA10, and SFMA20 was 10 ± 2, 7 ± 1, and 5 ± 1%, respectively. This can be explained by the chemical structure and conformation of SF, which contains three different conformations, namely, random coils, α-helixes (silk I), and β-sheets (silk II) (Figure S1).^6^ Previous research demonstrated that the structure of SF changes from a random coil/α-helix to a β-sheet structure as the concentration of the SF solution increases and the water content of the solution decreases.^72^ Lu et al. found that degradation rates of SF films were reversely correlated with the silk II content.^73^ The investigation conducted by Kambe et al. demonstrated a link between the β-sheet concentration and the degradation time of the SF.^68^ Increasing the fraction of β-sheets reduces SF’s enzymatic and hydrolytic degradation while increasing the scaffold stability and degradation time.^69,70^ After a period of 10 weeks, the quantity of degradation for SFMA5, SFMA10, and SFMA20 reaches 13 ± 3, 9 ± 1, and 7 ± 1%, respectively. As SFMA5 has a lower concentration than those of SFMA10 and SFMA20, there is a lower β-sheet content (Figure S1), resulting in a higher degradation rate. As a result, this could be a potential factor leading to an explanation of why SFMA5 shows a higher degradation rate than the other two SFMAs, SFMA10 and SFMA20.

The previous studies show that pristine HA is not completely degradable in a short period of time, as reported in earlier research; degradation is largely dependent on the incubation temperature and molecular weight.^74^ Adding methacrylate functional groups to HA using methacrylation agents like GMA^74^ and MA^23^ makes it more stable over time, which makes it a better choice for biomedical uses and drug delivery. Furthermore, DoM, molecular weight, and concentration of the modified HA have a significant impact on its degradation profile.^23,75^ According to the HAMA1 degradation results, after 5 weeks, 24 ± 0.5% of the HAMA1 has degraded, indicating enhanced stability compared to pristine HA. After a period of 10 weeks, the HAMA1 hydrogel exhibited a higher degradation rate in comparison to the SFMA hydrogel, which reached a rate of 42 ± 0.4%. Then, SFMA–HAMA degradation was investigated under the same experimental conditions to directly compare the mixture with those of the single components. By incorporation of HAMA1 into SFMA5, SFMA10, and SFMA20 matrices, the degradation rate was slightly modified. The results show that degradation percentages of SFMA5–HAMA1, SFMA10–HAMA1, and SFMA20–HAMA1 after 5 weeks were approximately 12 ± 3, 10 ± 4, and 9 ± 2%, respectively, which are slightly higher than those without HAMA. The degradation results of SFMA5–HAMA1, SFMA10–HAMA1, and SFMA20–HAMA1 at the end of 10 weeks were approximately 17 ± 3, 14 ± 4, and 13 ± 2%, respectively, which are higher than the degradation percentages for SFMA5, SFMA10, and SFMA20, which were approximately 13 ± 3, 8 ± 1, and 7 ± 1%, respectively. SF also showed a similar trend after methacrylation. In previous studies by Kim et al., it was found that SFMA degraded to approximately 25% of its initial weight after 30 days in PBS containing protease XIV.^28^ However, in the absence of the enzyme, the degradation was significantly reduced.^76^ These findings are consistent with the results of our studies.

Based on these results, it was demonstrated that it is possible to modify the degradation rate of SFMA–HAMA hydrogels. This could lead to the development of hydrogels with tailored degradation times, allowing, for example, better control over the release of embedded drugs or other therapeutic agents. In general, degradation behavior of SF varies depending on the preparation methods, structural factors, pore size, concentrations of silk fibroin, and host immune system response.^15,77^ Considering the low degradation ratio of both SFMA and SFMA–HAMA composite biomaterials, they can be considered as a suitable scaffold candidate for musculoskeletal tissue regeneration. The introduction of a methacrylate group on both SF and HA resulted in the creation of a reactive site, leading to covalent bonding in both monophasic and composite states in SFMA and HAMA networks in the presence of LAP and UV, with a range of physicochemical properties.

To compare our results with previous studies, we have prepared Table 2 that provides a comprehensive comparison between our research and those of the published studies. There are several key parameters included in the table, including the photoinitiator concentration, LAP or IQ concentration, DoM percentage, swelling ratio, compressive strength, and storage modulus. Our comparison provides a clearer understanding of our research’s advances and contributions based on the differences and similarities in the performance characteristics of the materials under study.

To assess the biomaterials’ overall biocompatibility and safety, *in vitro* cytotoxicity testing was conducted in accordance with ISO 10993.^49^ To determine cytotoxicity, distilled water is used as a positive control, while tissue culture plates were used as negative controls. Furthermore, the NIH3T3 cells have previously been demonstrated to be suitable for the evaluation of cytotoxicity.^78^ Wataha et al. found that NIH3T3 cells (also used in this study) had a similar level of cytotoxicity to primary cells.^78−80^ To attain a better, more reliable outcome, we applied both direct contact and extract dilution methods to samples from HAMA, SFMA, and SFMA–HAMA using NIH3T3 cell culture. For the assessment of the cytotoxicity of the HAMA, SFMA, and SFMA–HAMA hydrogels, NIH3T3 cells were exposed to the extracted medium at dilution factors of 0, 4, and 10 for 24 h. Figure S3A presents the cytotoxicity effect of the various HAMA, SFMA, and SFMA–HAMA hydrogels on the NIH3T3 cells. As stated in ISO 10993-5:2009, a cell viability below 70% of the blank represents a cytotoxic effect.^49,81^ All groups of samples have shown greater than 70% cell viability regardless of dilution factors, indicating suitable cytocompatibility and the absence of strong toxicity. In the undiluted sample, HAMA showed a viability of 75 ± 3%. With an increase in concentration from 5 to 10 wt % and 20 wt %, SFMA showed increased viability of 77 ± 6, 93 ± 8, and 99 ± 12%, respectively. This could be explained by an increase in the β-sheet content as the SFMA concentration was increased from 5 wt % to 20 wt % (Figure S1). Numata et al. demonstrated that the cell viability of human mesenchymal stem cells (hMSCs) grown on SF significantly improved as the concentration of SF increased, possibly due to the increasing β-sheet content, elastic modulus, network size, and bound water content.^82,83^

The addition of HAMA1 to the SFMA matrix enhanced the cytocompatibility of the hydrogel. By incorporating HAMA into the SFMA5 hydrogel at a dilution factor of 0, cell viability was improved from 77 ± 6 to 99 ± 7%. Additionally, this trend was observed for both SFMA10 and SFMA20 following the incorporation of HAMA. Regardless of the specific dilution factors, all SFMA–HAMA composite hydrogels exhibit >95% cell viability. In the study carried out by Garcia-Fuentes et al., it was examined how HA affects the molecular conformation of SF, in particular its transition into a crystalline β-sheet form.^84^ Their findings indicate that the introduction of HA into the SF network induces the formation of β-sheets within the SF-HA structure.^84^ Consistent with the previous study, the addition of HAMA1 to the SFMA20 network resulted in an increase in the sheet content from 73.3% to 86.9% (Figure S1). The reason for the observed results can be attributed to the introduction of HAMA into SFMA, facilitating the crystallization process and induceing the formation of β-sheets within the SFMA–HAMA blend structure.^84^ Based on prior research, it has been confirmed that an increase in β-sheet content could contribute to a higher cell viability in hMSCs.^82,83^ It can be concluded that the increased quantity of β-sheets enhances both the structural integrity and the biocompatibility of the material, leading to improved cellular viability.

In addition, cytocompatibility was analyzed by direct contact with the biomaterials (Figure S3B). Direct contact cytotoxicity evaluation is a highly sensitive and reliable method for detecting low levels of cytotoxicity.^85^ In the direct cytotoxicity assay, HAMA1 showed a cell viability of 78 ± 6%, whereas for SFMA5, SFMA10, and SFMA20, the viability was 85 ± 7, 88 ± 2, and 73 ± 11%, respectively. Adding HAMA to the SFMA network and producing SFMA5–HAMA1, SFMA10–HAMA1, and SFMA20–HAMA1 composites improved the cell viability to 88 ± 3, 85 ± 0.4, and 85 ± 4%, respectively. According to the obtained data, all samples displayed a higher percentage of cell viability than 70%, thus indicating that they are nontoxic according to ISO 10993-5:2009.^49^

**Figure 8:**
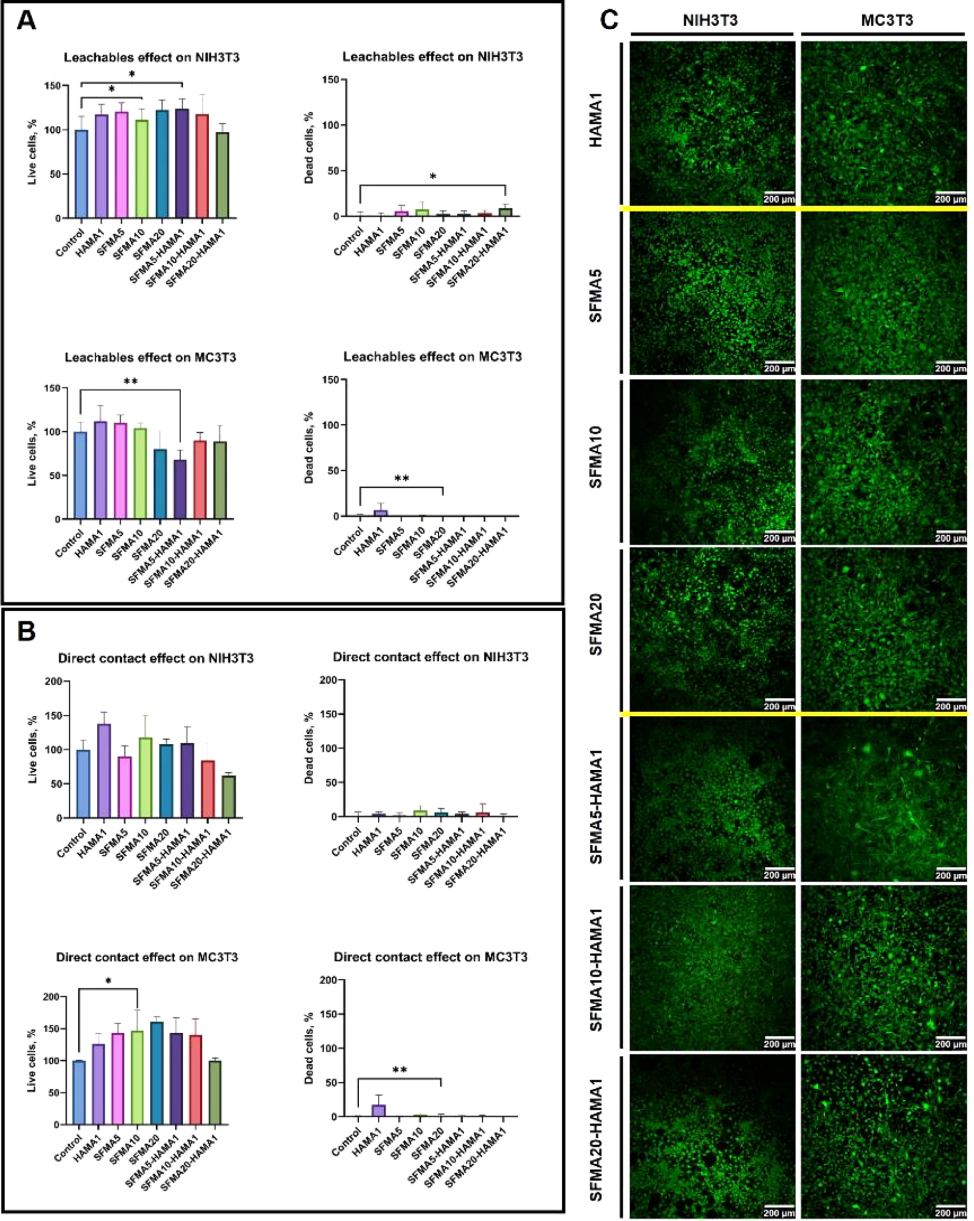
Results of live/dead assays on fibroblasts (NIH3T3) and preosteoblasts (MC3T3) and the (A) effects of the potential leachables (*n* = 6) and (B) direct contact with the materials (*n* = 3) were analyzed with one-way ANOVA followed by Dunnett’s multiple comparison test. Statistically significant differences versus control cells are indicated with *p* < 0.05 and *p < 0.01. (C) Representative confocal microscopy images of NIH3T3 and cells after 24 h of incubation in direct contact with the synthesized materials display the typical morphologies of NIH3T3 cells. Live cells exhibiting active metabolism were labeled with calcein-AM and appear in green, whereas the nuclei of dead cells were labeled with propidium iodide, which displays red fluorescence. The cells were maintained in direct contact with SFMA5, SFMA10, SFMA20, HAMA1, SFMA5–HAMA1, SFMA10–HAMA1, and SFMA20–HAMA1.

NIH3T3 and MC3T3 are two distinct cell lines commonly used to evaluate biomaterial interactions in the contexts of wound healing and bone tissue engineering, respectively.^86,87^ Moreover, we assessed the cytotoxicity of our materials and their interactions with these cell lines through a live/dead assay (Figure 8A–C), providing valuable insights into their biocompatibility and therapeutic potential. Live/dead assay results showed that all synthesized materials were biocompatible (Figure 8A–C). Interestingly, our results indicate that potential leachables from the materials stimulated the growth of NIH3T3 fibroblasts (Figure 8C), whereas direct contact stimulated the growth of MC3T3 preosteoblasts (Figure 8B). This is because stiffness is a critical parameter in the interaction between the cell and the materials. Preosteoblasts proliferated more rapidly on stiff substrates. It is important to note, however, that stiffness does not affect the proliferation of fibroblast cells.^86^ Although the number of dead cells was statistically greater than that of the positive controls (SFMA20–HAMA1 for extracts on NIH3T3, HAMA1 for both extracts, and direct contact on MC3T3), the percentages of dead cells were only 9%, 7%, and 17%, respectively. Due to the nature of the material, it was challenging to manipulate the discs in HAMA1 samples with forceps in a controlled manner without disturbing the cells in direct contact. It can therefore be hypothesized that the higher percentage of dead cells observed is a consequence of the physical damage caused by manual manipulation of the HAMA1 material.

Furthermore, we evaluated the viability and spreading of two types of cell lines, including NIH3T3 and MC3T3 in direct contact with SFMA, HAMA, and SFMA–HAMA hydrogels. A representative live–dead image of NIH3T3 cells obtained by confocal microscopy after 24 h of incubation in direct contact with SFMA, HAMA, and SFMA–HAMA hydrogels is presented in Figure 8C. These images provide an insight into the typical morphology of NIH3T3 cells under these conditions, as well as the cytocompatibility of the materials used. The results indicate that the cells have spread well on the scaffold and are alive with a low number of dead cells. In Figure 8C, live–dead images of MC3T3 cells are presented after 24 h of incubation with SFMA, HAMA, and SFMA–HAMA hydrogels. Furthermore, MC3T3 cells showed comparable viability to NIH3T3, suggesting that HAMA, SFMA, and SFMA–HAMA hydrogels are biocompatible.

## 4 Conclusions

In this work, we introduced a composite hydrogel made of semisynthetic derivatives of SF and HA. A systematic study was carried out to investigate the effects of different compositions on the rheological, mechanical, swelling, and degradation properties. The introduction of the methacrylate group on both SF and HA facilitates photo-cross-linking and enhances their mechanical properties, controlled gelation, and structural integrity. When SFMA and HAMA are mixed under conditions that help polymerization happen, covalent bonds are made between their methacrylate groups. This network has a unique set of properties because it combines the mechanical strength of SFMA with the bioactivity and hydrophilicity of HAMA. This research also shows that changing the amount of SFMA to HAMA can change the cross-linking density and network structure, which lets the mechanical and biological properties of the hydrogel to be tailored to specific needs. Accordingly, the combination of SFMA and HAMA to form a composite hydrogel enables the creation of an innovative composite hydrogel ink, which presents a number of benefits over using either SFMA or HAMA individually. These advantages include higher mechanical strength, elasticity, degradation rate, and biocompatibility in comparison to hydrogels produced using solely SFMA or HAMA. Materials were confirmed cytocompatible based on cell viability testing using direct contact live–dead and leachable assays performed on NIH3T3 and MC3T3 cell lines. Furthermore, the combination of SF and HA resulted in synergistic properties and the possibility of tuning the composition toward desired applications. Based on the promising results of stress–strain and cyclic loading tests, SFMA and SFMA–HAMA hydrogels can be potentially used in biomedical settings. The stiffer versions of the composite hydrogel introduced here have significant potential to expand applications, e.g., in cartilage engineering, whereas softer formulations could be used, for example, in connective tissue regeneration.

## Supporting Information Available

Quantification of the secondary structures from FTIR measurements, additional dynamic mechanical properties data, and cytotoxicity assessment by MTT assay (PDF)
