# Supplementary material for: Preparation and Characterization of Photo-Cross-Linkable Methacrylated Silk Fibroin and Methacrylated Hyaluronic Acid Composite Hydrogels
Source: Biomacromolecules. 2024 Oct 14;25(11):7078–97. doi: 10.1021/acs.biomac.4c00319 (PMC11558566; doi:10.1021/acs.biomac.4c00319)
Supplement: Supplementary file 1 — bm4c00319_si_001.pdf [file bm4c00319_si_001.pdf]

Preparation and characterization of photocrosslinkable methacrylated silk fibroin and methacrylated hyaluronic acid composite hydrogels – Supporting Information

*Jhaleh Amirian[1,2 *], Jacek K. Wychowaniec[3], Matteo D'Este[3], Andrea J. Vernengo[3,δ], Anastasija Metlova[4], Antons Sizovs[4,2], Agnese Brangule[1,2*], and Dace Bandere[1,2*]*

[1] Department of Pharmaceutical Chemistry, Riga Stradins University, Riga, LV-1007, Latvia

[2] Baltic Biomaterials Centre of Excellence, Headquarters at Riga Technical University, Riga, LV-1048, Latvia

[3]AO Research Institute Davos, Clavadelerstrasse 8, 7270 Davos, Switzerland

[4] Laboratory of Pharmaceutical Pharmacology, Latvian Institute of Organic Synthesis, Riga, LV-1006, Latvia

[δ] Current address: Department of Chemical and Biomedical Engineering, Henry M. Rowan College of Engineering, Rowan University, Glassboro, New Jersey, 08028, USA

[*]**Corresponding authors** Jhaleh Amirian, (jalehamirian@gmail.com), Agnese Brangule (agnese.brangule@rsu.lv) and Dace Bandere (dace.bandere@rsu.lv)

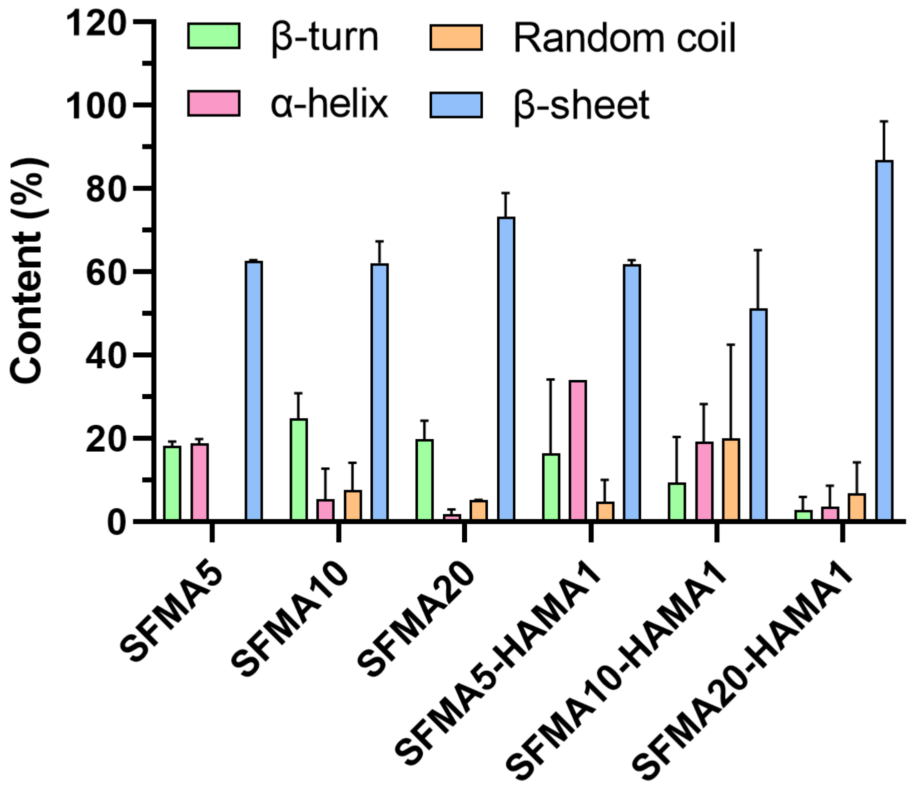

Figure S1. *Quantifying the β-turn, random coil, α-helix, and β-sheet content of various SFMA (5, 10, 20 wt.%) and SFMA (5, 10, 20 wt.%)-HAMA1 hydrogel secondary structures using fitted amide I spectra. Amide I (1600 cm^{-1} - 1700 cm^{-1}) was used for the fitting procedure.*

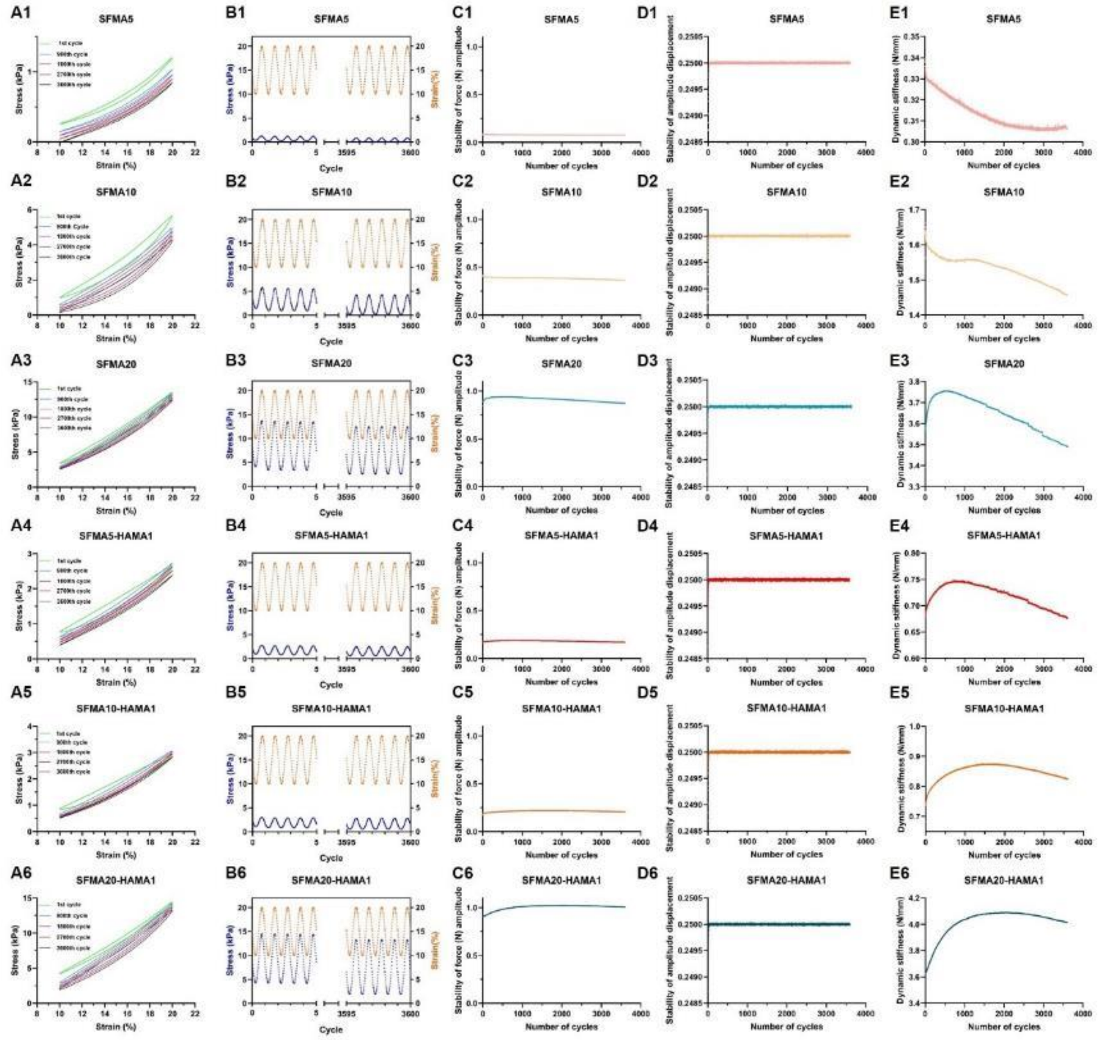

Figure S2. *Mechanical analysis.* ***(A)*** *Cyclic compressive stress-strain loading and unloading curves of SFMA5****(A1)****, SFMA10* ***(A2)****, SFMA20* ***(A3)****, SFMA5-HAMA1****(A4)****, SFMA10-HAMA1****(A5)****, and SFMA20-HAMA1(A6) hydrogels on 1st, 900th, 1800th, 2700th, 3600th cycles.* ***(B)*** *compressive cycle-stress curve and cycle-strain curve for repeated loading up to 20% strain of SFMA5* ***(B1)****, SFMA10* ***(B2)****, SFMA20* ***(B3)****, SFMA5-HAMA1* ***(B4)****, SFMA10-HAMA1* ***(B5)****, and SFMA20-HAMA1* ***(B6)*** *hydrogels at first (1 to 5 cycle) and last five cycles (3595-3600 cycle).* ***(C)*** *Stability of the force amplitude in N versus cycles from zero to 3600 cycle of SFMA5* ***(C1)****, SFMA10* ***(C2)****, SFMA20* ***(C3)****, SFMA5-HAMA1* ***(C4)****, SFMA10-HAMA1* ***(C5)****, and SFMA20-HAMA1****(C6)*** *hydrogels.* ***(D)*** *Stability of the amplitude displacement in N versus cycles from zero to 3600 cycle of SFMA5* ***(D1)****, SFMA10* ***(D2)****, SFMA20* ***(D3)****, SFMA5-HAMA1* ***(D4)****, SFMA10-HAMA1* ***(D5)****, and SFMA20-HAMA1* ***(D6)*** *hydrogels.* ***(E)*** *Toughness curves (Force to displacement in N/mm) corresponding to number of cycles SFMA5* ***(E1)****, SFMA10* ***(E2)****, SFMA20* ***(E3)****, SFMA5-HAMA1* ***(E4)****, SFMA10-HAMA1* ***(E5)****, and SFMA20-HAMA1* ***(E6)*** *hydrogels.*

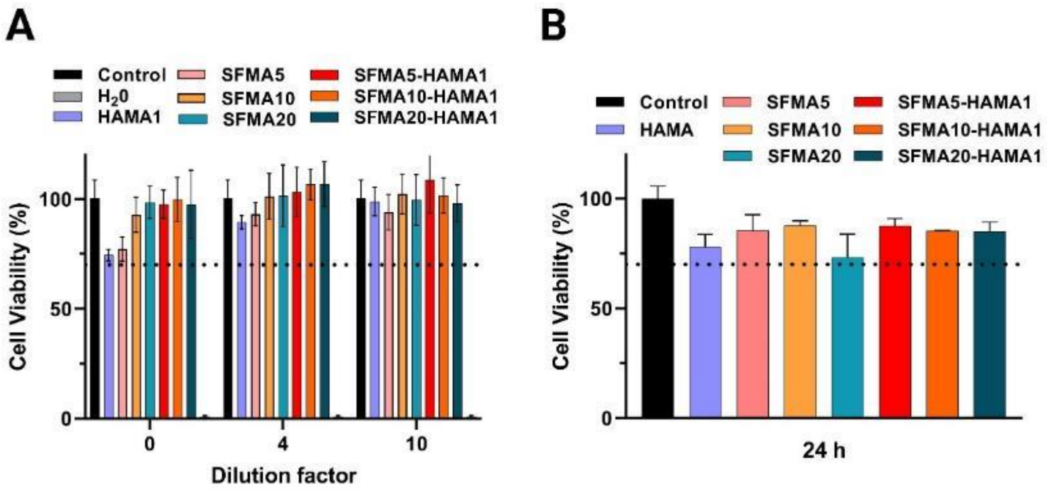

Figure S3. *Cytocompatibility assay:* ***(A)*** *indirect cytotoxicity showing* NIH3T3 *cell viability using different dilution factors (dilution 0: 100% extract, dilution 4= 25% extract solution and dilution factor 10: 10% extract solution) and* ***(B)*** NIH3T3 metabolic activity in *direct contact of HAMA 1%, SFMA (5, 10, and 20%) and SFMA5-HAMA1, SFMA 10-HAMA1, and SFMA20-HAMA1 hydrogels by MTT assay after 24h. Tissue culture plate used as control and H_2O were used as negative and positive control for cytotoxicity assays.*
